# Revision of the *Agrilus occipitalis* species–group (Coleoptera, Buprestidae, Agrilini)

**DOI:** 10.3897/zookeys.256.4272

**Published:** 2013-01-03

**Authors:** Eduard Jendek

**Affiliations:** 1 Canadian Food Inspection Agency, K. W. Neatby Bldg., 960 Carling Avenue, Ottawa, Ontario, K1A 0C6, Canada

**Keywords:** Buprestidae, Agrilini, *Agrilus*, new species, synonymy, lectotype designation, *Citrus*, pest

## Abstract

The *Agrilus occipitalis* species–group is redefined and diagnosed. Two species from this group, *Agrilus auriventris* Saunders, 1873 and *Agrilus occipitalis* (Eschscholtz, 1822), are known as the serious pests of cultivated *Citrus* trees. Overall twenty-three taxa are included in the *Agrilus occipitalis* species–group. A complete list of references, type material, species examined and distribution is given for each taxon. The host plants, adult occurrence and altitude range is cited for most taxa. Habitus of all taxa and aedeagi of available males are pictured. Images of primary type specimens are provided. A character state matrix table for diagnostic characters is given for all taxa to facilitate their determination.

The following new taxonomic and nomenclatural acts are proposed. **New species**:eight new species are described: *Agrilus mucidus* **sp. n.**, *Agrilus nebulosus* **sp. n.**, *Agrilus picturatus* **sp. n.**, *Agrilus pluvius* **sp. n.**, *Agrilus pseudoambiguus* **sp. n.**, *Agrilus tesselatus* **sp. n.**, *Agrilus trepanatus* **sp. n.** and *Agrilus umrongso* **sp. n.** **Proposed** **synonyms**: eight synonyms are proposed: *celebicola* Obenberger, 1924, **syn. n.** (synonym of *occipitalis* Eschscholtz, 1822); *connexus* Kerremans, 1900, **syn. n.** (synonym of *occipitalis* Eschscholtz, 1822); *cupricauda* Saunders, 1867 **syn. n.** (synonym of *occipitalis* Eschscholtz, 1822); *evinadus* Gory & Laporte, 1839, **syn. n.** (synonym of *occipitalis* Eschscholtz, 1822); *nirius* Obenberger, 1924 syn. reconfirmed (synonym of *occipitalis* Eschscholtz, 1822); *oblatus* Kerremans, 1900, **syn. n.** (synonym of *occipitalis* Eschscholtz, 1822); *samoensis* Blair, 1928, **syn. n.** (synonym of *auriventris* Saunders, 1873); *tebinganus* Obenberger, 1924, **syn. n.** (synonym of *occipitalis* Eschscholtz, 1822). **New lectotype designations**: six lectotypes are designated: *Agrilus celebicola* Obenberger, 1924; *Agrilus cupricauda* Saunders, 1867; *Agrilus korenskyi* Obenberger, 1923; *Agrilus kurandae* Obenberger, 1923; *Agrilus nirius* Obenberger, 1924; *Agrilus nitidus* Kerremans, 1898.

## Introduction

This publication presents the first comprehensive revision of the *Agrilus* taxa of the *occipitalis* species–group. The group was established and defined by Jendek and Grebennikov (2011) for seven species distributed in East Asia. With another sixteen taxa, including the eight new added in this work, the overall number of species of this group reaches twenty-three.

The state of species taxonomy has remained unrevised despite the fact that the two most serious pests in the citrus orchards *Agrilus occipitalis* (Eschscholtz, 1822) and *Agrilus auriventris* Saunders, 1873 belong to this group. Most of the species from the *Agrilus occipitalis* species–group are distributed in South and Eastern Asia but some spread well beyond this area: *Agrilus diversornatus* Jendek, 2011 to Russian Far East; *Agrilus occipitalis* and *Agrilus biakanus* Curletti, 2006 to Australasia. The occurrence of the chronic *Citrus* pest *Agrilus auriventris* in Polynesia and *Agrilus occipitalis* in Micronesia is most likely an introduction.

## Material and methods

The format of the taxonomic part, style of the new species descriptions and morphological terms follow those used in Jendek and Grebennikov (2011). Type data, type images and examined material published in Jendek and Grebennikov (2011) are omitted.

According to Article 74.7.3 of the ICZN (1999), lectotype designations after 1999 “*must contain an express statement of the taxonomic purpose of the designation*”. Lectotype desig- nations herein are provided in order to preserve the stability of nomenclature by fixing the status of the specimen as the sole name-bearing type of a particular nominal taxon. Lectotype designations were made with careful attention to previously accepted usage of a name.

**Abbreviations for collections**

**BMNH** The Natural History Museum, London, United Kingdom

**EJCB** Collection of E. Jendek, Bratislava, Slovak Republic [currently in Ottawa, Canada]

**IZAS** Institute of Zoology, Academia Sinica, Beijing, China

**MNHN** Muséum national d’Histoire Naturelle, Paris, France

**NHMB** Naturhistorisches Museum, Basel, Switzerland

**NMPC** National Museum (Natural History), Prague, Czech Republic

**NSMT** National Science Museum (Natural History), Tokyo, Japan

**USNM** National Museum of Natural History, Washington D. C. , USA

**ZIN** Zoological Institute, Russian Academy of Sciences, St. Petersburg, Russia

## Taxonomy

### *Agrilus occipitalis* species–group

**Diagnosis**. Medium (>5 mm) or large species (>10 mm), body shape cuneiform or parallel. Head usually impressed medially with curved sculpture at both sides; eyes small or moderate. Pronotum with anteromedial, posteromedial and lateral impressions; rarely without apparent impressions. Prehumerus carinal or filamentary; rarely tubercular or obsolete. Elytra monochromatic or with elytral apices carmine; apices separately arcuate, rarely separately subtruncate; elytral pubescence fasciate or ornamental, rarely absent. Prosternal process flat, sides subparallel or dilated, rarely narrowed. Sexual dimorphism not obvious except for the longer ventral pubescence in male.

**Distribution**.East, South and Southeast Asia, Indonesia, Australasia, Oceania.

**Host plants**. Adoxaceae (*Sambucus*), Rutaceae (*Citrus*, *Euodia*, *Fagara*, *Zanthoxylum*), Rosacae (*Sorbaria*).

Within the *Agrilus occipitalis* species–group seven subgroups can be recognized (Appendix):

**A: elytral apices separately subtruncate**

***Agrilus tesselatus***-subgroup

Species: *Agrilus tesselatus* sp. n.

Diagnostic characters: antennae very short, serrate from antennomere 5; pronotum obviously convex, without apparent impressions, maximum width of pronotum at posterior margin; groove on apex of last ventrite deeply sinuate; ovipositor square.

**B: elytral apices separately subarcuate**

**B1:** species with elytral apex pubescent and prosternal lobe predominantly emarginate.

***Agrilus perroti***-subgroup

Species: *Agrilus perroti* Descarpentries & Villiers, 1963, *Agrilus umrongso* sp. n. and *Agrilus zanthoxylumi* Li Meng Lou, 1989.

Diagnostic characters: body large or medium sized; head with deep medial longitudinal impressions; maximum width of pronotum at posterior margin, rarely at middle; elytral pubescence at least partly fasciate; apex of elytra pubescent and concolor with elytral disk; ovipositor prolonged.

***Agrilus auroapicalis***-subgroup

Species: *Agrilus auroapicalis* Kurosawa, 1957, *Agrilus diversornatus* Jendek, 2011, *Agrilus ishigakianus* Tôyama, 1985.

Diagnostic characters: body medium sized; head with deep medial longitudinal impressions; anterior pronotal lobe obvious; pronotum widest at posterior margin or at middle; prehumerus long, reaching to anterior pronotal angles, often joined with pronotal marginal carina; elytral pubescence at least partly fasciate; apex of elytra with strikingly different color than disk; prosternal lobe emarginate; apex of pygidium arcuate; groove on apex of last ventrite arcuate; ovipositor prolonged.

**B2:** species with prosternal lobe subtruncate or arcuate, elytral apex glabrous (except for *Agrilus yamawakii*) and prosternal process often dilated.

***Agrilus inamoenus***-subgroup

Species: *Agrilus inamoenus* Kerremans, 1892, *Agrilus mucidus* sp. n., *Agrilus pluvius* sp. n., *Agrilus tonkineus* Kerremans, 1895

Diagnostic characters: body medium or large, robust; head with deep medial longitudinal impressions; pronotum widest at posterior margin or at middle; elytral pubescence mosaic or at least partly mosaic; apex of pygidium angulate or with short protrusion, rarely arcuate; apex of last ventrite smooth, without medial carinula; ovipositor square.

***Agrilus ambiguus*-subgroup**

Species: *Agrilus ambiguus* Kerremans, 1895, *Agrilus picturatus* sp. n., *Agrilus pseudoambiguus* sp. n.

Diagnostic characters: body medium sized, slender; head with medium or deep medial impressions; antennae long; pronotum widest in middle, rarely at anterior margin; elytral pubescence mosaic or at least partly mosaic; pygidium angulate, with protrusion or long spine; ovipositor prolonged.

***Agrilus auriventris*-subgroup**

Species: *Agrilus alesi* Obenberger, 1935, *Agrilus auriventris* Saunders, 1873, *Agrilus nebulosus* sp. n., *Agrilus trepanatus* sp. n., *Agrilus yamawakii* Kurosawa, 1957.

Diagnostic characters: body medium or large, robust; head with or without medial impression; pronotum widest at anterior margin or in middle, rarely at posterior margin; elytral pubescence fasciate or ornamental, rarely missing; elytral apex concolor with elytral disk, rarely indistinctly carmine; ovipositor prolonged.

***Agrilus occipitalis*-subgroup**

Species: *Agrilus biakanus* Curletti, 2006, *Agrilus horniellus* Obenberger, 1935, *Agrilus occipitalis* (Eschscholtz, 1822), *Agrilus sordidulus* Obenberger, 1916.

Diagnostic characters: body medium, robust; head rarely without medial impression; pronotum widest in middle; elytral pubescence fasciate or ornamental, rarely missing; pygidium arcuate or with short protrusion, never with long spine; ovipositor prolonged.

### Alphabetical list of species

#### Agrilus alesi

Obenberger, 1935

http://species-id.net/wiki/Agrilus_alesi

Fig. 11 (habitus) ; Fig. 31 (aedeagus) ; Fig. 41 (pygidium)

Agrilus alesi Obenberger, 1935 (*Agrilus*) Obenberger 1935b: 164 (description) – Miwa and Chûjô 1936: 15 (catalog; Japan) – Obenberger 1936a: 940 (world catalog) – Kurosawa 1963: 152 (subspecies of *auriventris*; characters; Japan) – Chûjô 1970: 20 (subspecies of *auriventris*; checklist; faunal records; Japan (Loo-Choo Archipelago)) – Kurosawa 1974: 2-3 (characters; notes) – Tôyama 1985a: 24 (iconography; Japan) – Hirashima 1989: 322 (checklist; Japan) – Morimoto and Tadauchi 1995: 231 (checklist; Japan) – Akiyama and Ohmomo 1997: 28-29 (checklist; Japan: Ryukyus (Okinawa)) – Jendek 2006: 396 (Palaearctic catalog) – Bellamy 2008: 1960 (world catalog) – Jendek and Grebennikov 2011: 38-39 (lectotype designation; synonymy; references; faunal records; distributional summary; East Asia). = *Agrilus sacchari* Obenberger, 1940 (*Agrilus*) Obenberger 1940: 175-176 (description) – Miwa and Chûjô 1940: 74 (cited as *sachari*; faunal records; Okinawa: Loo-Choo) – Kurosawa 1974: 3 (subspecies of *alesi*; characters; notes) – Hirashima 1989: 322 (subspecies of *alesi*; checklist; Japan) – Peng Zhongliang 1994: 19 (faunal records; China) – Morimoto and Tadauchi 1995: 231 (subspecies of *alesi*; checklist; Japan) – Akiyama and Ohmomo 1997: 29 (subspecies of *alesi*; checklist; Japan) – Hua Li Zhong 2002: 90 (checklist; China: Sichuan) – Jendek 2006: 396 (subspecies of *alesi*; Palaearctic catalog) – Bellamy 2008: 1960 (subspecies of *alesi*; world catalog) – Jendek and Grebennikov 2011: 39 (synonym of *alesi*; lectotype designation). = *Agrilus aritai* Tôyama, 1985 (*Agrilus*) Tôyama 1985b: 42-44 (description) – Tôyama 1985a: 25 (iconography; Japan) – Hirashima 1989: 322 (checklist; Japan) – Morimoto and Tadauchi 1995: 231 (checklist; Japan) – Akiyama and Ohmomo 1997: 29 (checklist; Japan: Ryukyus (Ishigaki-jima I.)) – Jendek 2006: 396 (Palaearctic catalog) – Bellamy 2008: 1976 (world catalog) – Jendek and Grebennikov 2011: 39 (synonym of *alesi*).

##### Type material.

*Agrilus alesi* Obenberger, 1935. Type locality. Loo-choo: Okinawa. Lectotype designated by Jendek and Grebennikov (2011).

*Agrilus sacchari* Obenberger, 1940. Type locality. Insulae Loo-Choo; Okinawa. Lectotype designated by Jendek and Grebennikov (2011).

*Agrilus aritai* Tôyama, 1985. Type locality. Hirano, Ishigakijima Is. Holotype exa-mined by Jendek and Grebennikov (2011).

##### Diagnosis.

Size 7.2–8.5 mm. *Agrilus alesi* can be distinguished from closely related *Agrilus auriventris* by the smaller size; by the more transverse pronotum with the maximum width in the middle; by the obvious anterior pronotal lobe and by the pygidium with the long spine on apical margin (Fig. 41). See also Appendix.

##### Additional material.

CHINA: 2 ♂, 1 ♀ (USNM): “CB [citrus borer?] adults, Chengtu, China, v.28.-vi.5. ‘[19]39, Kovlieu [Note: Chengtu may refer to many po- pulated places in several provinces of China”. JAPAN: Kyushu: 23 (USNM): “Kagoshima, Kyushu, 5-v-1940, F. Yano”; 2 ♂, 1 ♀ (USNM): “Kagoshima: Kyushu, Japan, 5-v-1940 (F. Yano)”. For further records see Jendek and Grebennikov (2011).

##### Adult occurrence:

5–6–7–9.

##### Host plant.

*Citrus*: Akiyama and Ohmomo (1997)**.**

##### Distribution.

CHINA[provincial level unknown]. JAPAN: Kyushu; Ryukyu islands (Okinawa incl.).

#### Agrilus ambiguus

Kerremans, 1895

http://species-id.net/wiki/Agrilus_ambiguus

Fig. 8 (habitus) ; Fig. 28 (aedeagus)

Agrilus ambiguus Kerremans, 1895 (*Agrilus*) Kerremans 1895: 220-221 (description) – Kerremans 1903: 282 (catalog) – Jakobson 1913: 798 (synonym of *ambiguellus*; catalog) – Obenberger 1936a: 1072 (world catalog) – Jendek 2000: 502 (valid species; notes) – Jendek 2006: 396 (Palaearctic catalog) – Bellamy 2008: 1962 (world catalog) – Jendek 2012: 3 (lectotype designation). = *ambiguellus* Kerremans, 1903 (*Agrilus*; unnecessary replacement name) Kerremans 1903: 282 (unnecessary replacement name proposal) – Jakobson 1913: 798 (catalog; Russia and Europe) – Obenberger 1936a: 1072 (world catalog) – Kurosawa 1974: 3 (characters; notes) – Jendek 2000: 502 (synonym of *ambiguus*) – Jendek 2006: 396 (synonym of *ambiguus*; Palaearctic catalog) – Bellamy 2008: 1962 (synonym of *ambiguus*; world catalog).

##### Type material.

*Agrilus ambiguus* Kerremans, 1895. Type locality. Sikkim: Kurseong. Lectotype designated by Jendek (2012).

##### Diagnosis.

Size 6.7–10 mm. *Agrilus ambiguus* differs from the closely related *Agrilus picturatus* sp. n. and *Agrilus pseudoambiguus* sp. n. by having the head only feebly impressed medially; by the black vertex and by having elytral apices concolor with elytral disk (See also Appendix).

##### Additional material.

INDIA: Assam: 1 ♀ (EJCB): “NE India, Assam, 1999, 5 km N of Umrongso, 700m, 25°27'N, 92°43'E, 17.-25.v., Dembický & Pacholátko leg.”. Meghalaya: 7 (EJCB): “NE India, Meghalaya state, West Garo Hills, Nokrek Nat.Park, 9–17. V. 1996 alt.1100+150m, GPS N25°29.6', E90°19.5' (WGS 84), E. Jendek & O. Šauša leg.”; 7 (EJCB): “NE India, Meghalaya, 1400 m, Nokrek N. P. , 3 km S Daribokgiri, 25°27'N, 90°19'E, 26.iv.1999, Dembický & Pacholátko leg.”. 1 ♂ (EJCB): “NE India, Meghalaya, 1400 m, Nokrek N. P. , 3 km S Daribokgiri, 25°27'N, 90°19'E, 26.iv.1999, J. Rolčík leg.”. West Bengal: 2 (NMPC): “Darjeeling [labeled as pescheti Obnb.; nomen nudum]”. LAOS: Louang Namtha: 1 ♂, 2 ♀ (EJCB): “Laos, Louang Namtha pr., 21°09'N, 101°19'E, Namtha–Muang Sing, 5-31.v.1997, 900-1200 m, Vit Kubáň leg.”. Phongsali: 1 ♂ (EJCB): “Lao-N, Phongsaly prov., 21°41'-2'N, 102°06'–08'E, 28.v.-20.vi.2003 Phongsaly env., ~1500m, Vít Kubáň leg.”; 1 ♂ (EJCB): “Lao, Phongsaly prov. 21°41'N, 102°06'E Phongsaly env. 6–17.v.2004, 1500 m, P. Pacholátko leg.”.

##### Adult occurrence:

4–5–6. **Altitude range**: 700–1500 m.

##### Host plant.

Unknown.

##### Distribution.

INDIA: Assam; Meghalaya; Sikkim; West Bengal. LAOS: Louang Namtha; Phongsali.

#### Agrilus auriventris

Saunders, 1873

http://species-id.net/wiki/Agrilus_auriventris

Fig. 12 (habitus) ; Fig. 32 (aedeagus); Fig. 42 (pygidium)

Agrilus auriventris Saunders, 1873 (*Agrilus*) Saunders 1873: 517 (description) – Samouelle 1819: 9-17 [not seen] (biology; biocontrol; Japan) – Lewis 1879: 15 (catalog; Japan) – Kerremans 1885: 152 (catalog) – Schönfeldt 1887: 113 (*Anambus*; catalog; Japan) – Kerremans 1892: 248 (catalog) – Kerremans 1903: 282 (catalog) – Jakobson 1913: 798 (catalog; Russia and Europe) – Obenberger 1926: 659 (Palaearctic catalog) – Tanaka 1928: 1437-1444 [not seen] (pest; Japan) – Miwa 1931: 126 (catalog; Formosa) – Ogloblin and Reichardt 1932: 277 (pest) – Yuasa 1932: 653 (characters; Japan) – Yuasa 1933: 281 (larva) – Miwa and Chûjô 1936: 15 (catalog; Japan) – Obenberger 1936a: 958 (world catalog) – Chûjô and Matuda 1940: 65 (checklist; faunal records; Japan (Kyushu)) – Miwa 1940: 72 (checklist; Japan) – Yuasa 1949: 128 (characters; Japan) – Kurosawa 1950: 1115 – Mühlmann 1954: 83 (pest in Asia; biology) – Iga 1955: 78 (iconography; Japan) – Ter Minasyan 1955: 450 (notes) – Kurosawa 1956: 1115 (characters; Japan) – Shimizu et al. 1961: 57-59 (biology) – Yamamoto et al. 1961: 1-8 [not seen] (biology; pest) – Balachowsky et al. 1962: 286 (economic importance) – Iga 1962: 78 (iconography; Japan) – Isrigaya 1963: 351-355 (biology; larva; economic importance; treatment; Japan (Wakayama)) – Ohgushi 1963: 92-96 (pest; ovary and pre-oviposition period; Japan) – Woo Sheh Ming 1964: 61-71 (larval biology; protection; China: Chekiang) – Ohgushi 1966b: 361-366 [not seen] (biology; ecology; Japan) – Ohgushi 1966a: 55-63 (biology) – Yoshikawa et al. 1969: 178 (notes) – Guryeva 1974: 98 (pest of agriculture cultures; references) – Kurosawa 1974: 2, 3 (characters; notes) – Ohgushi 1978: 62-73 (population ecology; Japan: Nagasaki prefecture) – Tôyama 1985a: 24 (iconography; Japan) – Peng Zhongliang 1987: 354 (checklist; China) – Hirashima 1989: 322 (checklist; Japan) – Peng Zhongliang 1992: 398-399 (characters; notes; Hunan) – Morimoto and Tadauchi 1995: 231 (checklist; Japan) – Akiyama and Akiyama 1996: 184 (faunal records; Japan: Honshu) – Naka and Ohashi 1996: 43-44 (occurrence; chemical control; Japan: Honshu) – Akiyama and Ohmomo 1997: 29-30 (checklist; Japan) – Hua Li Zhong 2002: 89 (checklist) – Lin Gui Rui 2002: 233 (checklist; pest) – Peng Zhongliang 2002: 268 (characters; Fujian) – Jendek 2006: 396 (Palaearctic catalog) – Shi Fu Ming et al. 2006: 724 (notes) – Wei et al. 2006: 302-308 (larval instars and characters; China: Zheijang) – Zheng et al. 2006: 806 (spatial distribution pattern; attacking strategy; China) – Huangfu et al. 2007: 682-688 (ovarian development; ovariole; oviposition duration; fecundity) – Wei et al. 2007: 79-84 (immature stages; chorion; hatching; pupation) – Bellamy 2008: 1985 (world catalog) – Hill 2008: 279, 539 (pest; control; life history) – Jendek and Grebennikov 2011: 46-47 (lectotype designation; synonymy; references; types; diagnosis; faunal records; host plants; distributional summary; East Asia). = *graptelytrus* Obenberger, 1914 (*Agrilus*) Obenberger 1914: 43 (in Czech), 48-49 (in German) (description) – Obenberger 1926: 654 (Palaearctic catalog) – Obenberger 1936a: 1084 (world catalog) – Descarpentries and Villiers 1963: 104, 118 (species incertae sedis) – Bellamy 2008: 2116 (world catalog) – Jendek and Grebennikov 2011: 46 (synonym of *auriventris*; lectotype designation). = *fleutiauxi* Bourgoin, 1922 (*Agrilus*) Bourgoin 1922: 23 (description) – Théry 1935a: 132 (faunal record; China) – Obenberger 1936a: 1083 (world catalog) – Descarpentries and Villiers 1963: 108, 118 (characters; faunal records; Tonkin; Formose; Chine: Hong-Kong, Kiangsi; Birmanie) – Jendek 2006: 398 (Palaearctic catalog) – Bellamy 2008: 2096 (world catalog) – Jendek and Grebennikov 2011: 46, 47 (synonym of *auriventris*; lectotype designation). = *pidjinus* Obenberger, 1924 (*Agrilus*; cited as *podjinus* on page 53 and as *pidjinus* on page 58) Obenberger 1924b: 53-54, 58 (description; [Note: Multiple original spelling: Precedence of the name *pidjinus* has been fixed by the original author (Obenberger 1926) as the first reviser (Article 24.2.4)]) – Obenberger 1926: 654 (Palaearctic catalog) – Obenberger 1936a: 1022 (world catalog) – Peng Zhongliang 1987: 357 (checklist; China) – Hua Li Zhong 2002: 90 (checklist; China: Hongkong) – Jendek 2006: 400 (Palaearctic catalog) – Bellamy 2008: 2235 (world catalog) – Jendek and Grebennikov 2011: 46, 47 (synonym of *auriventris*; lectotype designation). = *samoensis* Blair, 1928 (*Agrilus*), **syn**. **n.**Blair 1928: 108-109 (description) – Théry 1934: 148 – Obenberger 1936a: 1101 (world catalog) – Bellamy 2008: 2278 (world catalog). **Unavailable names** = *citri* Matsumura Quayle 1938: 314 (biology; Formosa; [Note: Quayle attributed this name to Matsumara [= Matsumura] but his use of the name was not found and Quayle presented no characters]) – Fang Zhigang and Wu Hong 2001: 80 (checklist; China: Zhejiang) – Hua Li Zhong 2002: 89 (synonym of *auriventris*) – Lin Gui Rui 2002: 233 (checklist; pest) – Jendek and Grebennikov 2011: 236 (unavailable name).

##### Type material.

*Agrilus auriventris* Saunders, 1873. Type locality. Japan. Lectotype designated by Jendek and Grebennikov (2011).

*Agrilus graptelytrus* Obenberger, 1914. Type locality. China, Tonkin: Phu-long-thuan. Lectotype designated by Jendek and Grebennikov (2011).

*Agrilus fleutiauxi* Bourgoin, 1922. Type locality. not given [Note: Indo-Chine française is cited in the title of the publication]. Lectotype designated by Jendek and Grebennikov (2011).

*Agrilus pidjinus* Obenberger, 1924. Type locality. China: Hong-Kong. Lectotype designated by Jendek and Grebennikov (2011).

*Agrilus samoensis* Blair, 1928. Type locality. Upolu: Apia; Malololelei. Holotype (Fig. 61), ♀, (BMNH): “Type H. T. [p] [round label with red border] \ Samoan Is. [p] Upolu Malololelei 2000 ft, 28.xi.1924 [h] P. A. Buxton and G. H. Hopkins [p] \ Agrilus Samoensis Type Blr. [h] frt. K. G. Blair [p]”. Paratypes: 1 paratype (MNHN); 1 paratype (BPBM). Described from 4 specimens (holotype, paratypes).

##### Diagnosis.

Size: 5.0–8.8 mm. *Agrilus auriventris* can be distinguished from the most close *Agrilus nebulosus* sp. n. by the pygidium which is angulate or armed with a short protrusion (Fig. 42). See also Appendix.

##### Additional material.

CHINA: Guangxi: 1 (IZAS): “Guangxi, Longzhou, 140m, 1.v.1963, Y. Wang leg.”. VIETNAM: 1 (IZAS): “Tonkin, Hoa Binh, vii.1931, A. De Cooman leg.”. For further records see Jendek and Grebennikov (2011).

##### Adult occurrence:

4–5–6–7–8–10. **Altitude range:** 140–700 m.

##### Host plant.

*Citrus*sp. For the detailed bibliography see Jendek and Grebennikov (2011).

##### Distribution.

CHINA: Fujian; Guangdong; Guangxi; Hong Kong; Hubei; Hunan; Jiangxi; Sichuan; Taiwan; Zhejiang. JAPAN: Honshu; Kyushu. LAOS. MYANMAR. SAMOA. VIETNAM.

#### Agrilus auroapicalis

Kurosawa, 1957

http://species-id.net/wiki/Agrilus_auroapicalis

Fig. 5 (habitus) ; Fig. 46 (Holotype)

Agrilus auroapicalis Kurosawa, 1957 (*Agrilus*) Kurosawa 1957: 190–191 (description) – Kurosawa 1974: 3-4 (characters; notes) – Tôyama 1985a: 24 (iconography; Japan) – Akiyama and Ohmomo 1997: 30 (checklist; Japan: Ryukyus; Taiwan) – Hua Li Zhong 2002: 89 (Kerremans is cited as the author; checklist; China: Taiwan) – Mühle 2003: 46 (checklist; Taiwan) – Jendek 2006: 396 (Pala-earctic catalog) – Bellamy 2008: 1985 (world catalog) – Jendek 2012: 6 (synonymy). = *laurenconi* Descarpentries & Villiers, 1963 (*Agrilus*) Descarpentries and Villiers 1963: 104, 109 (description) – Bellamy 2008: 2161 (world catalog) – Jendek 2012: 6 (synonym of *laurenconi*).

##### Type material.

*Agrilus auroapicalis* Kurosawa, 1957. Type locality. Mt. Nanjin-zan, Formosa. Holotype (Fig. 46) examined by Jendek (2012).

*Agrilus laurenconi* Descarpentries & Villiers, 1963. Type locality. Tonkin: Hoa-Binh. Holotype (Fig. 55) examined by Jendek (2012).

**Figures 1–12.**
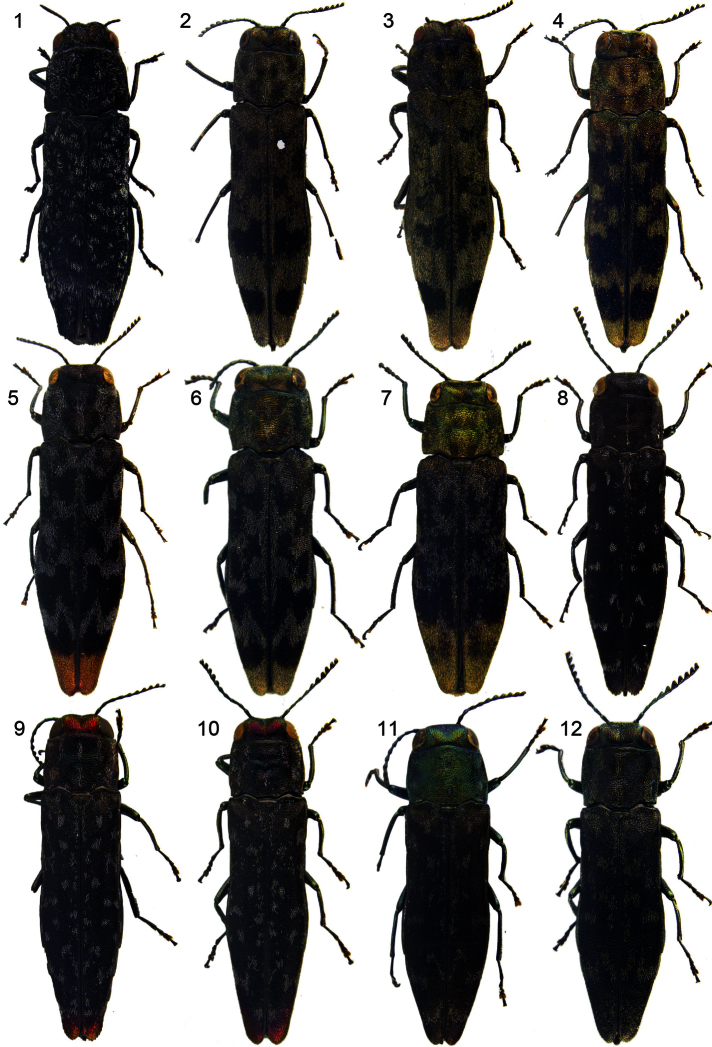
Habitus of *Agrilus*: **1** *Agrilus tesselatus* sp. n – Holotype **2** *Agrilus perroti* Descarpentries & Villiers, 1963 **3** *Agrilus umrongso* sp. n – Holotype **4** *Agrilus zanthoxylumi* Li Meng Lou, 1989 **5** *Agrilus auroapicalis* Kurosawa, 1957 **6** *Agrilus auroapicalis ishigakianus* Tôyama, 1985 **7** *Agrilus diversornatus* Jendek, 2011 – Holotype **8** *Agrilus ambiguus* Kerremans, 1895 **9** *Agrilus picturatus* sp. n – Holotype **10** *Agrilus pseudoambiguus* sp. n – Holotype **11** *Agrilus alesi* Obenberger, 1935 **12** *Agrilus auriventris* Saunders, 1873.

##### Diagnosis.

Size 5.8–8.1 mm. *Agrilus auroapicalis* differs from the similar *Agrilus diversornatus* mainly by the larger eyes, by pronotum widest in the middle and by pronotal lobe obviously arcuate. See also Appendix.

##### Additional material.

CHINA: Guizhou: 1 ♀ (MNHN): “Kouy-Tchéou, R. P. J. R. Chaffanjon 1903”. Taiwan: 1 (EJCB): “Meiyuan, Nantou Hsien, Taiwan, 5.v.1993, Luo Chinchi leg.”. LAOS: Louangphrabang: 1 ♀ (EJCB): “Laos-N, 23.iv.1999, Louangphrabang prov. 20°42'N, 102°54'E, 25 km E Muang Ngoy, 1000 m, Vít Kubáň leg.”. VIETNAM: Gia Lai: 1 (EJCB): “S Vietnam, Gia Lai-Kon Tum pr., 5 km N Ankhé, 19.x.1979”; 1 (EJCB): “Vietnam, Gialai, Contum Tram Cap, 20.4.1995, Gorochov”.

##### Adult occurrence:

4–5–10. **Altitude range:** 1000 m.

##### Host plant.

Unknown.

##### Distribution.

CHINA: Guizhou; Taiwan. LAOS: Louangphrabang. VIETNAM: Gia Lai; Hoa Binh.

#### Agrilus auroapicalis ishigakianus

Tôyama, 1985

http://species-id.net/wiki/Agrilus_auroapicalis_ishigakianus

Fig. 6 (habitus) ; Fig. 27 (aedeagus)

Agrilus ishigakianus Tôyama, 1985 (*Agrilus*; subspecies of *auroapicalis*) Tôyama 1985a: 33-34 (description) – Hirashima 1989: 322 (subspecies of *auroapicalis*; checklist; Japan) – Morimoto and Tadauchi 1995: 231 (subspecies of *auroapicalis*; checklist; Japan) – Akiyama and Ohmomo 1997: 30 (subspecies of *auroapicalis*; checklist; Japan: Ryukyus (Ishigaki-jima I.)) – Jendek 2006: 396 (subspecies of *auroapicalis*; Palaearctic catalog) – Bellamy 2008: 1985 (subspecies of *auroapicalis*; world catalog) – Fukutomi and Kurihara 2011: 27 (subspecies of *auroapicalis*; faunal record; biology; Ryukyu islands).

##### Type material.

*Agrilus auroapicalis ishigakianus* Tôyama, 1985. Type locality. Mt. Omotodake, Ishigaki-jima Isl. Types not examined. See Remarks. Described from 3 specimens (holotype, alotype, paratype).

##### Diagnosis.

Size 6.1 mm; it can be distinguished from *Agrilus auroapicalis auroapicalis* by having the body smaller, less produced apically; by the golden-brown dorsal color; by the ornamental elytral pubescence more extensive mostly along the suture and by having the color of elytral apices less contrasting to that of the nominal subspecies. See also Appendix.

##### Additional material.

JAPAN: Ryukyu islands: 1 ♂ (EJCB): “1996.4.19, Ishigaki Is., Okinawa, Ryukyu, K. Takahashi leg.”.

##### Host plant.

*Euodia meliifolia*: Fukutomi and Kurihara (2011).

##### Distribution.

JAPAN: Ryukyu islands (Okinawa incl.).

##### Remarks.

The type specimens of this taxon were not studied; they should be preserved in NSMT as stated by Tôyama (1985a). The taxonomic concept was judged from the specimen determined by S. Ohmomo.

#### Agrilus biakanus

Curletti, 2006

http://species-id.net/wiki/Agrilus_biakanus

Fig. 47 (habitus of holotype)

Agrilus biakanus Curletti, 2006 (*Agrilus*; subgenus *Agrilus*) Curletti 2006: 178–179, 220 (description) – Bellamy 2008: 1996 (subgenus *Agrilus*; world catalog).

##### Type material. 

*Agrilus biakanus* Curletti, 2006. Type locality. Mokmer, Biak Isl., N. G. Type specimens were not examined. Image of the holotype (Fig. 47) was adopted from Curletti (2006). See also Remarks. Described from 10 specimens (holotype, paratypes).

##### Diagnosis. 

Size: 6.9–9.2 mm. This species is distinctive by the glabrous elytra (Curletti 2006). See also Appendix.

##### Host plant.

Unknown.

##### Distribution.

INDONESIA: Irian Jaya.

##### Remarks.

No specimens of this species were available for this study. The assignment of this taxon to *Agrilus occipitalis* species–group is based on the original description and on the image of aedeagus which is very similar to that of *Agrilus occipitalis*.

#### Agrilus diversornatus

Jendek, 2011

http://species-id.net/wiki/Agrilus_diversornatus

Fig. 7 (habitus of holotype)

Agrilus diversornatus Jendek, 2011 (*Agrilus*) Jendek In: Jendek and Grebennikov 2011: 89–90, 267 (description).

##### Type material.

*Agrilus diversornatus* Jendek, 2011. Type locality. Eastern Russia, south Primorskiy kray, Lazovskii zapovednik, kordon Korpad’, 43°15'17"N, 134°07'59"E. Holotype (Fig. 7) examined by Jendek and Grebennikov (2011).

##### Diagnosis.

Size 6.8–7.1 mm. From the similar *Agrilus auroapicalis* it differs mostly by the smaller eyes; by pronotum widest at the posterior margin and by the pronotal lobe being obviously angulate. See also Appendix.

##### Additional material.

Known only from type specimens.

**Adult occurrence:** 7–8.

##### Host plant.

*Sorbaria*: Jendek and Grebennikov (2011).

##### Distribution.

RUSSIA: Primorskiy kray.

#### Agrilus horniellus

Obenberger, 1935

http://species-id.net/wiki/Agrilus_horniellus

Fig. 16 (habitus) ; Fig. 35 (aedeagus)

Agrilus horniellus Obenberger, 1935 (*Agrilus*; replacement name for *horni* Théry not Kerremans) Obenberger 1935a: 121 (replacement name proposal) – Obenberger 1936b: 92 (erronoeusly cited as new replacement name) – Obenberger 1936a: 1085 (world catalog) – Bellamy 2008: 2127 (world catalog) – Jendek 2012: 9 (synonymy). = *horni* Théry, 1904 (*Agrilus*; [preoccupied]) Théry 1904: 161-162 (description) – Obenberger 1935a: 121 (synonym of *horniellus*) – Obenberger 1936b: 92 (synonym of *horniellus*) – Obenberger 1936a: 1085 (synonym of *horniellus*) – Bellamy 2008: 2127 (synonym of *horniellus*; world catalog) – Jendek 2012: 9 (synonym of *horniellus*; lectotype designation).

##### Type material.

*Agrilus horniellus* Obenberger, 1935. Type locality. See: *Agrilus horni* Théry, 1904. See: *Agrilus horni* Théry, 1904.

*Agrilus horni* Théry, 1904. Type locality. Nalanda. Lectotype designated by Jendek (2012).

##### Diagnosis.

Size: 6.5–7.3 mm. *Agrilus horniellus* differs from the very similar *Agrilus occipitalis* by having the apex of pygidium arcuate and the apex of elytra concolor with the elytral disk. See also Appendix.

##### Additional material.

SRILANKA:1 ♂ (EJCB): “Sri Lanka: Anu Distr., 6 miles south of Tantirimalai, 2000ft, 31 Oct 1976”; 1 ♀ (EJCB): “Ceylon, E. Prov., Pottuvil, 1-12/vii.-1983 Ole Mehl. leg.”; 1 ♀ (EJCB): “Ceylon, N. C. Prov., Anuradhapura, 22-26/vi.-1985, Ole Mehl. leg.”.

##### Adult occurrence:

6–7–10. **Altitude range:** 610 m.

##### Host plant.

Unknown.

##### Distribution.

SRILANKA.

##### Remarks.

*Agrilus horniellus* may be conspecific with *Agrilus occipitalis*, but its original taxonomic concept was tentatively retained due to limited specimens available for examination.

#### Agrilus inamoenus

Kerremans, 1892

http://species-id.net/wiki/Agrilus_inamoenus

Fig. 21 (habitus) ; Fig. 38 (aedeagus)

Agrilus inamoenus Kerremans, 1892 (*Agrilus*)Kerremans 1892a: 824-825 (description) – Kerremans 1903: 286 (catalog) – Obenberger 1936a: 1086 (world catalog) – Baudon 1961: 74 (faunal records; Laos) – Descarpentries and Villiers 1963: 104, 109 (lectotype designation; characters; faunal data; Birmanie; Laos; Tonkin; Annam) – Baudon 1968: 131, 143 (characters in key; faunal records; Laos) – Peng Zhongliang and Huang Bangkan 1995: 98-101 (characters; biology; China: Fujian) – Hua Li Zhong 2002: 89 (checklist; China: Fujian [Note: Misidentification]) – Peng Zhongliang 2002: 270 (characters; Fujian) – Jendek 2003: 181–182 (remark on lectotype designation) – Bellamy 2008: 2136 (world catalog) – Jendek and Grebennikov 2011: 109–110 (references; types; diagnosis; faunal records; host plants; distributional summary; East Asia).

##### Type material.

*Agrilus inamoenus* Kerremans, 1892. Type locality. Carin Cheba, 900–1100 m. Lectotype designated by Descarpentries and Villiers (1963).

##### Diagnosis.

Size 7.8–9.8 mm. Within the subgroup, *Agrilus inamoenus* is distinctive by the short antennae, by the prosternal lobe absent or vague and by the tubercular prehumerus. See also Appendix.

##### Additional material.

CHINA: 1 (IZAS): “[China] Shuangjiang, vi.1953 [in Chinese]”. Yunnan: 1 (IZAS): “Yunnan Xishuangbanna Xiaomengyang, 850m, 9.vii.1957, S. Y. Wang leg. [in Chinese]”; 3 (IZAS): “Yunnan Xishuangbanna Menghun, 750m, 2–7.vi.1958, S. Y. Wang leg. [in Chinese]”; 1 (IZAS): “Yunnan Xishuangbanna Damenglong, 650m, 5.v.1958, X. W. Meng leg. [in Chinese]”. THAILAND: Chaiyaphum: 1 (EJCB): “Thailand, Chaiyaphum Tat Tone NP, near stream, 15°58.771'N, 102°02.397'E, Malaise trap, 5–12.vii.2006, T. Jaruphan and O. Budsawong leg.”. Chiang Rai: 1 ♂ (USNM): “Khun Tan Mts, N Siam 3000 ft, HM. Smith May [19]33”. VIETNAM: Lam Dong: 1 (MNHN): “Djiring, Annam, H. Perrot”. For further re- cords see also Jendek and Grebennikov (2011).

##### Adult occurrence:

4–5–6–7. **Altitude range:** 420–1600 m.

##### Host plant.

*Citrus*: Hua Li Zhong (2002); *Sambucusjavanica*: Baudon (1968).

##### Distribution.

CHINA: Fujian; Yunnan. LAOS: Borikhamxai; Khammouan; Louang Namtha; Louangphrabang; Savannakhet; Xaignabouri; Xiangkhoang. MYA-NMAR: Karen State. THAILAND: Chaiyaphum; Chiang Mai, Chiang Rai. VIETNAM: Binh Dinh; Gia Lai; Hoa Binh; Lam Dong; Son La.

#### Agrilus mucidus sp. n.

urn:lsid:zoobank.org:act:1C67DFF8-5BF1-416C-9F7F-CD0F18B685C5

http://species-id.net/wiki/Agrilus_mucidus

Fig. 22 (habitus of holotype) ; Fig. 39 (aedeagus)

##### Description.

BODY. Size: 9.6–10.7 mm (Holotype 10 mm).Shape: subparallel, Build: robust, Posterior tapering part: short with broad apex, Color (dorsally): unicolored, Sexual modifications in male: not apparent. HEAD.Size: very large, Medial impression: deep, Epistoma: raised above frons. *Vertex*: Shape: markedly convex, Sculpture elements: rugae, Sculpture shape: semispherical, Sculpture density: dense, Sculpture intensity: rough. *Eyes*: Size: small, Shape: markedly protruding head outline, Lower margin: in line or below with antennal socket, Medial orbit: converging ventrally or subparallel. *Antennae*: Length: long, Width: slender, Serration: from antennomere 4, Antennomere 7–10 (shape): with obvious collum. PRONOTUM.Shape: transverse, Sides: markedly arcuate or slightly arcuate, Maximal width: at middle, Anterior margin: subequal to posterior. *Anterior lobe*: Size: mode-rate, Shape: arcuate or subangulate, Position: at level with anterior angles. *Posterior angles*: Shape: acute or obtuse or rectangular, Apex: blunt or sharp. *Disk*: Conve-xity: flat, Impressions: medial and lateral, Medial impression: anteromedial and posteromedial, Lateral impressions (intensity): shallow, Lateral impression (size): wide. *Prehumerus*: Development: filamentary, rarely carinal, Shape: bisinuate, Extent: to 1/3 of pronotal length, rarely to 1/2 of pronotal length, Anterior end: distant from lateral carina, Posterior end: joined with posterior angle or margin, Arc: moderate or obvious. *Lateral carinae*: Convergence: moderately convergent, Junction: present, Narrowest point: at posterior 1/5-1/4 of marginal carina. ELYTRA.Color: monochromatic, Humeral carina: absent. *Apices*: Arrangement: separate, Shape: arcuate. *Pubescence*: Color: monochromatic, Character: homogenous or with patches or spots of denser pubescence, Extent: entire ornamental with indication of fasciae or entire ornamental. Tomentum: Spots (pattern): postmedial only. STERNUM. *Prosternal lobe*: Size: large, Distal margin: arcuate. *Prosternal process*: Shape: subparallel, Sides: arcuate, Angles: obtuse, Angles (tips): blunt, Disc: flat. ABDOMEN.Tomentum: absent or present. *Pygidium*: Apical margin: angulate. *Sternal groove*: Extent: on three apical ventrites, Shape on the apex of last ventrite: arcuate. LEGS. *Metatarsus*: Size to metatibia: distinctly shorter than metatibia. *Tarsomere 1*: Size to following tarsomeres: longer than 2-3 but shorter than 2-4. GENITALIA. *Aedeagus* (Fig. 39): Symmetry: symmetric, Shape: widest in basal part, rarely subparallel, Modifications: apex of medial lobe sharply pointed. *Ovipositor*: Shape: square (uritiform).

##### Diagnosis.

From the very close *Agrilus tonkineus*, it can be distinguished by the flat pronotum; by the bi-sinuate prehumerus and by the presence of transverse tomentose strip at apical third of elytra. See also Appendix.

##### Type locality.

China,Hainan, Baihualing, 19.018, 109.836, altitude 300 m.

##### Type material.

Holotype (Fig. 22), ♂, (EJCB): “China, Hainan, Baihualing, 19.018, 109.836, alt. 300 m, vi. 2008 [p]”. Paratypes: 1 paratype (EJCB), 3 paratypes (IZAS) from the same locality as holotype; 1 paratype (EJCB): “[China] Hainan Qiongzhong Baihuashan Mt, 27.v.1997, P. Y. Yu leg. [transcription from Chinese]”. 1 paratype (USNM), 1 paratype (EJCB): “Taichow, China, 1933”.

**Figures 13–24.**
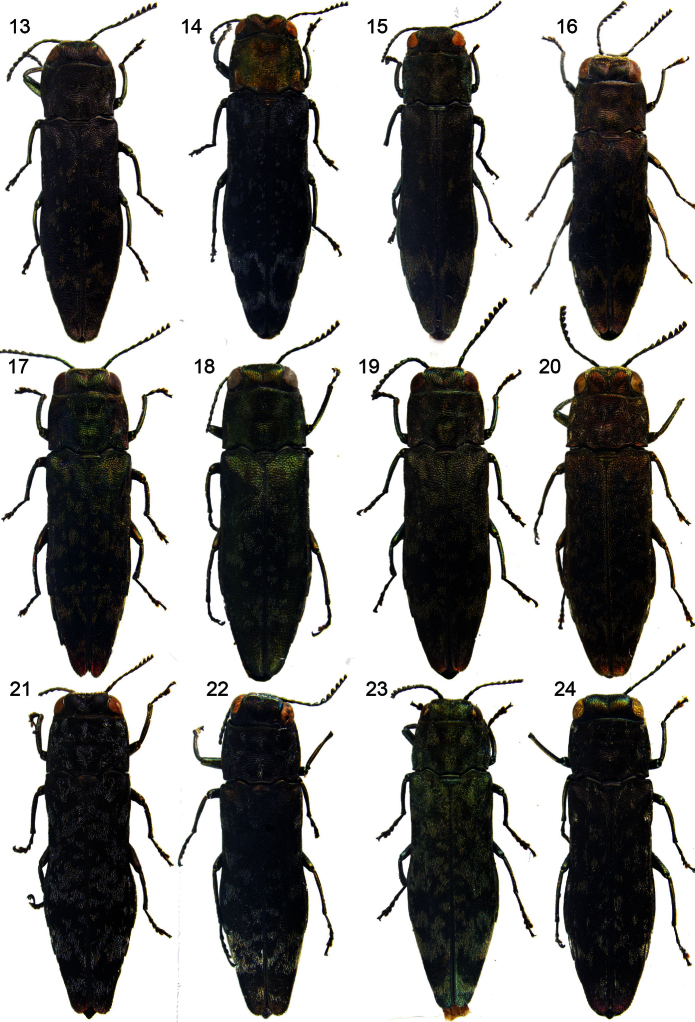
Habitus of *Agrilus*: **13** *Agrilus nebulosus* sp. n – Holotype **14** *Agrilus trepanatus* sp. n – Holotype **15** *Agrilus yamawakii* Kurosawa, 1957 **16** *Agrilus horniellus* Obenberger, 1935 **17** *Agrilus occipitalis* (Eschscholtz, 1822) – Laos, Vientiane, Ban Phabat **18** *Agrilus occipitalis* (Eschscholtz, 1822) – Papua New Guinea, Sideia island **19** *Agrilus occipitalis* (Eschscholtz, 1822) – Philippines, Palawan **20** *Agrilus sordidulus* Obenberger, 1916 **21** *Agrilus inamoenus* Kerremans, 1892 **22** *Agrilus mucidus* sp. n – Holotype **23** *Agrilus pluvius* sp. n – Holotype **24** *Agrilus sordidulus* Obenberger, 1916.

##### Adult occurrence:

5–6 . **Altitude range:** 300 m.

##### Host plant.

Unknown.

##### Distribution.

CHINA:Hainan; Zhejiang.

##### Etymology.

The specific name is Latin adjective *mucidus* (moldy). It refers to the elytral tomentum of the species.

#### Agrilus nebulosus sp. n.

urn:lsid:zoobank.org:act:2DF8A3BE-19D6-40D4-B183-D3EE333BF5F5

http://species-id.net/wiki/Agrilus_nebulosus

Fig. 13 (habitus of holotype) ; Fig. 33 (aedeagus)

##### Description.

BODY. Size: 6.4–7.5 mm (Holotype 6.5 mm).Shape: cuneiform, Posterior tapering part: long with narrow apex, Color (dorsally): unicolored, Sexual modifications in male: not apparent. HEAD. Medial impression: deep, rarely shallow, Epistoma: raised above frons, *Vertex*: Sculpture elements: rugae, Sculpture density: dense, *Eyes*: Size: moderate, Lower margin: in line or below with antennal socket, Medial orbit: converging ventrally, *Antennae*: Length: long, Width: slender, Serration: from antennomere 4. PRONOTUM. Shape: transverse, Sides: slightly arcuate, Maximal width: at anterior margin, Anterior margin: wider than posterior, *Anterior lobe*: Size: moderate, Shape: arcuate, Position: at level with anterior angles, *Posterior angles*: Shape: obtuse or rectangular, Apex: sharp, *Disk*: Impressions: medial and lateral, Medial impression: anteromedial and posteromedial, *Prehumerus*: Development: carinal, Shape: arcuate, Extent: to 1/3 of pronotal length, Anterior end: distant from lateral carina, Posterior end: joined with posterior angle or margin, Arc: moderate or weak, *Lateral carinae*: Convergence: moderately convergent, Junction: present, Narrowest point: at posterior 1/5-1/4 of marginal carina. ELYTRA. Color: monochromatic, Humeral carina: absent, *Apices*: Arrangement: separate, Shape: arcuate, *Pubescence*: Color: monochromatic, Extent: entire ornamental, rarely entire ornamental with indication of stripes. STERNUM. *Prosternal lobe*: Size: large, Distal margin: arcuate, *Prosternal process*: Size: wide, Shape: dilated, rarely subparallel, Sides: straight, Angles: acute, Angles (tips): blunt, Disc: flat, Projection (extend): distinctly beyond angles, *Mesosternum*: Mesosternal projection: flat. ABDOMEN. Tomentum: absent, *Pygidium*: Apical margin: arcuate, rarely angulate, *Sternal groove*: Extent: on all ventrites or on three apical ventrites, Shape on the apex of last ventrite: arcuate, rarely arcuately sinuate, Emargination (deepness): very shallow. LEGS. *Metatarsus*: Size to metatibia: distinctly shorter than metatibia, *Tarsomere 1*: Size to following tarsomeres: longer than 2-3 but shorter than 2-4 or subequal or longer than 2-4. GENITALIA. *Aedeagus* (Fig. 33): Symmetry: symmetric, Shape: widest in basal part, rarely subparallel, Modifications: apex of medial lobe sharply pointed, *Ovipositor*: Shape: markedly elongate.

**Figures 25–45.**
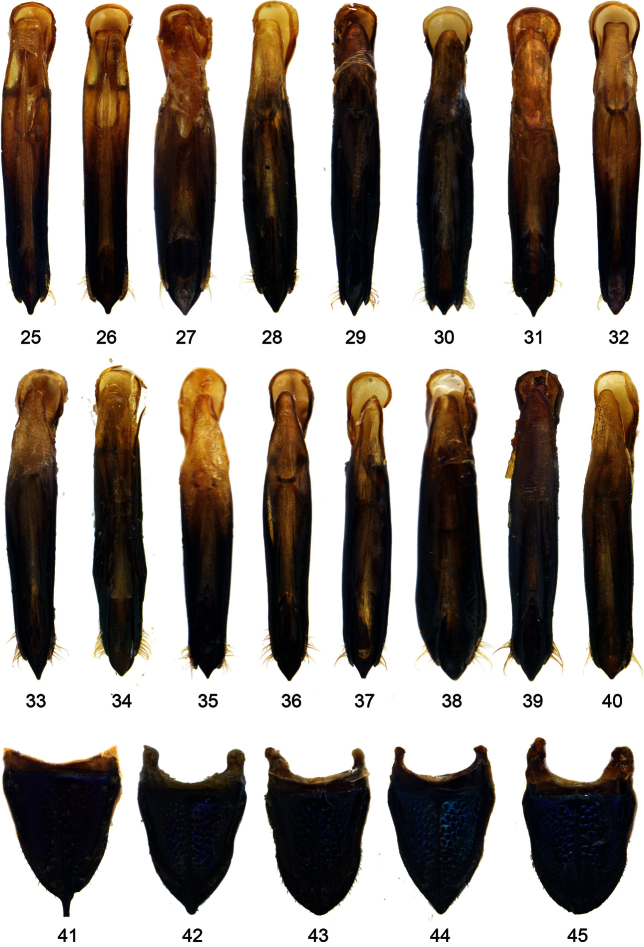
Aedeagus of *Agrilus*: **25** *Agrilus perroti* Descarpentries & Villiers, 1963 **26** *Agrilus zanthoxylumi* Li Meng Lou, 1989 **27** *Agrilus auroapicalis ishigakianus* Tôyama, 1985 **28** *Agrilus ambiguus* Kerremans, 1895 **29 ***Agrilus picturatus* sp. n – Holotype **30** *Agrilus pseudoambiguus* sp. n – Holotype **31** *Agrilus alesi* Obenberger, 1935 **32 ***Agrilus auriventris* Saunders, 1873 **33** *Agrilus nebulosus* sp. n – Holotype **34** *Agrilus yamawakii* Kurosawa, 1957 **35** *Agrilus horniellus* Obenberger, 1935 **36** *Agrilus occipitalis* (Eschscholtz, 1822) – Philippines, Palawan **37** *Agrilus sordidulus* Obenberger, 1916 **38** *Agrilus inamoenus* Kerremans, 1892 **39** *Agrilus mucidus* sp. n – Holotype **40** *Agrilus tonkineus* Kerremans, 1895.Pygidium of *Agrilus* **41** *Agrilus alesi* Obenberger, 1935 **42** *Agrilus auriventris* Saunders, 1873 **43** *Agrilus occipitalis* (Eschscholtz, 1822) – Laos, Vientiane, Ban Phabat **44** *Agrilus occipitalis* (Eschscholtz, 1822) – Papua New Guinea, Sideia island **45** *Agrilus occipitalis* (Eschscholtz, 1822) – Thailand, Mae Hong Son, Pai.

##### Diagnosis.

From the close *Agrilus auriventris*, it can be distinguished by the generally smaller size and slender body; by the pygidium arcuate apically and by the the groove on the apex of last ventrite arcuate (rarely sinuate). See also Appendix.

##### Type locality.

North Vietnam, 21°35N, 106°30E, 52 km southwest of Lang Son, altitude 370 m.

##### Type material.

Holotype (Fig. 13), ♂, (EJCB): “N Vietnam, 21°35N, 106°30E, 52 km SW of Lang Son, 27.iv.-6.v.1996, 370 m, Dembický and Pacholátko leg.”. Paratypes:1 ♂ paratype, 7 ♀ paratypes (EJCB) from the same locality as holotype.

##### Adult occurrence:

5–6 . **Altitude range**: 300 m.

##### Host plant.

Unknown.

##### Distribution.

VIETNAM:Lang Son.

##### Etymology.

The specific name is Latin adjective *nebulosus* (misty, hazy, indefinite, obscure). It refers to the faint ornamental elytral pubescence of the species.

#### Agrilus occipitalis

(Eschscholtz, 1822)

http://species-id.net/wiki/Agrilus_occipitalis

Figs 17–19 (habitus) ; Fig. 36 (Aedeagus); Figs 43–45 (pygidium) ; Fig. 60 (Lectotype)

Agrilus occipitalis (Eschscholtz, 1822) (*Buprestis*) Eschscholtz 1822: 79-80 (description) – Eschscholtz 1823: 135-136 (*Buprestis*) – Dejean 1833: 83 (catalog) – Dejean 1836: 93 (catalog) – Mannerheim 1837: 110 (notes) – Gemminger and Harold 1869: 1443 (catalog) – Saunders 1870: 23 (catalog) – Saunders 1871: 121 (catalog) – Saunders 1874: 323 (faunal records; Philippines) – Baer 1886: 126 (catalog; Philippines) – Kerremans 1892: 265 (catalog) – Kerremans 1903: 278 (catalog) – Schultze 1916: 57 (checklist; faunal records; Luzon) – Fisher 1921: 349, 356, 369 (checklist; Philippines) – Obenberger 1924c: 562 – Tan 1925: 583-584 [not seen] (pest; Philippines) – Fisher 1926: 242 – Clausen 1933: 29, 30 (pest) – Obenberger 1936a: 1094-1095 (world catalog) – Quayle 1938: 197-198, 314 (ecology; pest; control; Philippines) – Miwa and Chûjô 1940: 74 (faunal record; Formosa) – Mühlmann 1954: 84 (notes) – Balachowsky et al. 1962: 287 (pest on *Citrus*) – Macabasco 1964: 133-135 (biology; pest; control measures; Philippines: Luzon) – Anonymous 1969: 60 (pest; Papua New Guinea) – Yoshikawa et al. 1969: 178 (biological observation; Malaysia: Perak; Cambodia: Phnom-Penh) – Anonymous 1971: 189 (pest; Papua New Guinea) – Kurosawa 1974: 3 (characters; notes) – Jackman 1987: 28 (faunal records; Phillipines (Luzon)) – Hawkeswood and Turner 1994: 14-18 (biology; behaviour; Papua New Guinea) – Jendek 1998: 326 (lectotype designation) – Curletti 2006: 173-174, 222 (subgenus *Agrilus*; characters; faunal records; remarks; distributional summary; Indonesia: Maluku; New Guinea) – Jendek 2006: 400 (Palaearctic catalog) – Bellamy 2008: 2211-2212 (subgenus *Agrilus*; world catalog) – Hill 2008: 279, 539 (pest; control; life history). = *evinadus* Gory & Laporte, 1839 (*Agrilus*) **syn**. **n.**Gory and Laporte 1839: 30 (description) – Gray 1848: 36 (Buquet is cited as the author; checklist of taxa in the collection of the British museum) – Gemminger and Harold 1869: 1439 (cited as *evanidus*; catalog) – Saunders 1871: 121 (cited as *evanidaus*; catalog) – Kerremans 1892: 256 (cited as *evanidus*; catalog) – Kerremans 1903: 277 (cited as *evanidaus*; catalog) – Obenberger 1936a: 1082 (world catalog) – Descarpentries and Villiers 1963: 104, 110 (lectotype designation; characters; faunal records; remark; Cochinchine; Java) – Descarpentries and Chûjô 1968: 15 (faunal record; Vietnam) – Nelson and Bellamy 1993: 304 (authorship and publication date) – Jendek 1998: 321 (lectotype data) – Bellamy 1999: 3 (authorship assigned to Buquet) – Bellamy 2008: 2087 (world catalog). = *occipitalis* Gory, 1841 (*Agrilus*) Gory 1841: 222-223 (description; [Note: Gory’s name is based on the type specimen of Eschscholtz. The name *occipitalis* Gory is a junior objective synonym and a secondary homonym of *occipitalis* Eschscholtz]) – Nelson and Bellamy 1993: 304 (authorship and publication date) – Jendek 1998: 326 (synonym of *occipitalis* Eschscholtz; lectotype designation) – Curletti 2006: 173 (synonym of *occipitalis* Eschscholtz) – Jendek 2006: 400 (synonym of *occipitalis* Eschscholtz; Palaearctic catalog) – Bellamy 2008: 2212 (synonym of *occipitalis* Eschscholtz; world catalog). = *marmoreus* Deyrolle, 1864 (*Agrilus*) Deyrolle 1864: 146, 201–202 (description) – Gemminger and Harold 1869: 1442 (catalog) – Saunders 1871: 124 (catalog) – Kerremans 1892: 263 (catalog) – Kerremans 1903: 287 (catalog) – Obenberger 1936a: 1091 (world catalog) – Mühlmann 1954: 83 (notes) – Balachowsky et al. 1962: 287 (pest on *Citrus*) – Jendek 1998: 325 (lectotype designation) – Curletti 2001: 6, 21-22, 39 (subgenus *Agrilus*) – Bellamy 2002: 352 (subgenus *Agrilus*; catalog; Australia) – Williams 2002: 88 (faunal records; Australia) – Curletti 2006: 173 (synonym of *occipitalis* Eschscholtz) – Bellamy 2008: 2212 (synonym of *occipitalis* Eschscholtz; world catalog). = *cupricauda* Saunders, 1867 (*Agrilus*), **syn**. **n.**Saunders 1867: 520 (description) – Gemminger and Harold 1869: 1438 (catalog) – Saunders 1871: 124 (catalog) – Kerremans 1892: 253 (catalog) – Kerremans 1903: 284 (catalog) – Obenberger 1936a: 1079 (world catalog) – Bellamy 2008: 2049 (world catalog). = *nitidus* Kerremans, 1898 (*Agrilus*) Kerremans 1898: 179–180 (description) – Kerremans 1903: 277 (catalog) – Carter 1924a: 29 – Carter 1929: 276 – Obenberger 1936a: 1095 (presumed synonym of *occipitalis*; world catalog) – Théry 1936: 61 (synonym of *occipitalis*) – Carter 1940: 389 (synonym of *korenskyi*) – Curletti 2001: 21, 39 (synonym of *marmoreus*) – Bellamy 2002: 352 (synonym of *marmoreus*) – Curletti 2006: 173 (synonym of *occipitalis* Eschscholtz) – Bellamy 2008: 2212 (synonym of *occipitalis* Eschscholtz; world catalog). = *connexus* Kerremans, 1900 (*Agrilus*), **syn**. **n.**Kerremans 1900b: 5, 22, 28 (description) – Kerremans 1903: 278 (catalog) – Obenberger 1936a: 1078 (world catalog) – Bellamy 2008: 2035 (world catalog). = *oblatus* Kerremans, 1900 (*Agrilus*), **syn**. **n.**Kerremans 1900a: 340 (description) – Kerremans 1903: 278 (catalog) – Obenberger 1936a: 1094 (world catalog) – Jendek 2005: 14 (lectotype designation) – Bellamy 2008: 2206 (world catalog). = *korenskyi* Obenberger, 1923 (*Agrilus*) Obenberger 1923: 80-81 (description) – Carter 1924b: 536 (presumably conspecific with *semiviridis*) – Carter 1929: 276 (variety of *nitidus*) – Obenberger 1936a: 1089 (world catalog) – Carter 1940: 389 (synonymy) – Curletti 2001: 21, 39 (synonym of *marmoreus*; notes) – Bellamy 2002: 352 (synonym of *marmoreus*) – Curletti 2006: 173 (synonym of *occipitalis* Eschscholtz) – Bellamy 2008: 2212 (synonym of *occipitalis* Eschscholtz; world catalog). = *kurandae* Obenberger, 1923 (*Agrilus*) Obenberger 1923: 80 (description) – Carter 1924b: 536 (presumably near to *nitidus*) – Carter 1929: 276 (synonym of *nitidus*) – Obenberger 1936a: 1089 (world catalog) – Carter 1940: 389 (synonym of *korenskyi*) – Curletti 2001: 21, 39 (synonym of *marmoreus*) – Bellamy 2002: 352 (synonym of *marmoreus*) – Curletti 2006: 173 (synonym of *occipitalis* Eschscholtz) – Bellamy 2008: 2212 (synonym of *occipitalis* Eschscholtz; world catalog). = *celebicola* Obenberger, 1924 (*Agrilus*), **syn**. **n.**Obenberger 1924a: 123 (description) – Obenberger 1936a: 1077 (world catalog) – Bellamy 2008: 2019 (world catalog). = *nirius* Obenberger, 1924 (*Agrilus*), **syn**. **reconfirmed**Obenberger 1924a: 123-124 (description) – Théry 1927: 34 (synonym of *occipitalis*) – Obenberger 1931: 36 (subspecies of *occipitalis*) – Théry 1935c: 256 (synonym of *occipitalis*) – Obenberger 1936a: 1095 (subspecies of *occipitalis*; world catalog) – Kalshoven 1951: 697-699 (variety of *occipitalis*; larva; larval galleries; biology; Philippines; Java) – Mühlmann 1954: 84 (variety of *occipitalis*; notes on biology) – Bellamy 2008: 2212 (subspecies of *occipitalis* Eschscholtz; world catalog). = *tebinganus* Obenberger, 1924 (*Agrilus*), **syn**. **n.**Obenberger 1924a: 120 (description) – Obenberger 1936a: 1105 (world catalog) – Bellamy 2008: 2324 (world catalog). **Unavailable names** = *evanidus* Buquet Dejean 1836: 93 (catalog; [Note: Dejean attributed this name to Buquet but his use of the name was not found and Dejean presented no characters]) – Bellamy 2008: 2087 (unavailable synonym of *evinadus*; world catalog). = *evanidus* Gemminger & Harold Gemminger and Harold 1869: 1439 (Gory and Laporte are cited as authors; [Note: Incorrect subsequent spelling; Gemminger and Harold did not propose the name as new, they misspelled *evinadus* Gory & Laporte]) – Obenberger 1936a: 1082 (synonym of *evinadus*) – Descarpentries and Villiers 1963: 110 (synonym of *evinadus*) – Bellamy 2008: 2087 (unavailable synonym of *evinadus*; world catalog).

##### Type material.

*Buprestis occipitalis* Eschscholtz, 1822. Type locality. Auf der Inßel [= Insel] Luzon, bei Manilla. Lectotype (Fig. 60) designated by Jendek (1998).

*Agrilus evinadus* Gory & Laporte, 1839. Type locality. Java. Lectotype (Fig. 51) designated by Descarpentries and Villiers (1963).

*Agrilus occipitalis* Gory, 1841. Type locality. Iles Philippines. Lectotype designated by Jendek (1998).

*Agrilus marmoreus* Deyrolle, 1864. Type locality. I. Mysole et Batchian. Lectotype (Fig. 56) designated by Jendek (1998).

*Agrilus cupricauda* Saunders, 1867. Type locality. Penang. **Lectotype by present designation** (Fig. 50), ♀, (BMNH): “Type H. T. [p] [round label with red border] \ Penang [h] [oval blue label] \ Penang (Lamb.) Pascoe Coll. [p] \ Agrilus cupricauda Typ ES [h]”. Described from unknown number of syntypes.

*Agrilus nitidus* Kerremans, 1898. Type locality. Australie: Cocktown. **Lectotype by present designation** (Fig. 57), ♀, (BMNH): “Syn-Type [p] [round label with blue border] \ Cocktown Stauding [h] \ nitidus Kerr. Type [h] \ A. nitidus Kerrem. Australie [h] \ Kerremans 1903-59 [p]”. **Secondary**: 1 paralectotype (BMNH). Described from unknown number of syntypes.

*Agrilus connexus* Kerremans, 1900. Type locality. not given [Sumatra, Hindrapoera is cited in the title and introductory text]. Holotype by monotypy (Fig. 49), ♂, (BMNH): “Type [p] [round label with red border] \ Sumatra Weyers [h] \ connexus Kerr. Type [h] \ Kerremans 1903-59 [p] \ A. connexus Kerrem. Sumatra [h]”. Described from 1 specimen.

*Agrilus oblatus* Kerremans, 1900. Type locality. Sumatra. Lectotype (Fig. 59) designated by Jendek (2005).

*Agrilus korenskyi* Obenberger, 1923. Type locality. Australia. **Lectotype by present designation** (Fig. 53), ♂, (NMPC): “Australia [h] \ Typus [p] [red label] \ A. Kořenskyi m. Type [h] Det. Dr. Obenberger [p]”. Described from unknown number of syntypes.

*Agrilus kurandae* Obenberger, 1923. Type locality. Kuranda (Quensland). **Lectotype by present designation** (Fig. 54), ♀, (NMPC): “Kuranda Queensland [h] \ Typus [p] [red label] \ Agrilus kurandae m. Type [h] Det. Dr. Obenberger [p]”. Described from unknown number of syntypes.

*Agrilus celebicola* Obenberger, 1924. Type locality. Celebes. **Lectotype by present designation** (Fig. 48), ♀, (NMPC): “Drs. Sarasin S. O. Celebes Kolaka [p] [yellow label] \ TYPUS [p] [red label] \ Agrilus celebicola m. Type [h] Det. Dr. Obenberger [p]”. Described from unknown number of syntypes.

*Agrilus nirius* Obenberger, 1924 Type locality. Java; Ins. Batoe; Tanah Masa. **Lectotype by present designation** (Fig. 58), ♀, (NMPC): “Java Buitenzorg [h] \ Typus [p] [red label] \ Agrilus Nirius m. Type [h] Det. Dr. Obenberger [p]”. Secondary: 6 paralectotypes (NMPC); 1 paralectotype (RMNH). Described from unknown number of syntypes.

*Agrilus tebinganus* Obenberger, 1924. Type locality. Ostsumatra. **Lectotype by present designation** (Fig. 63), ♀, (NMPC): “Sumatra [h] \ Typus [p] [red label] \ tebinganus Kerr. n. sp. type \ Agrilus tebinganus m. Type [h] [Obenberger’s MS] Det. Dr. Obenberger [p]”. Secondary: 1 paralectotype (MNHN). Described from unknown number of syntypes.

**Figures 46–63.**
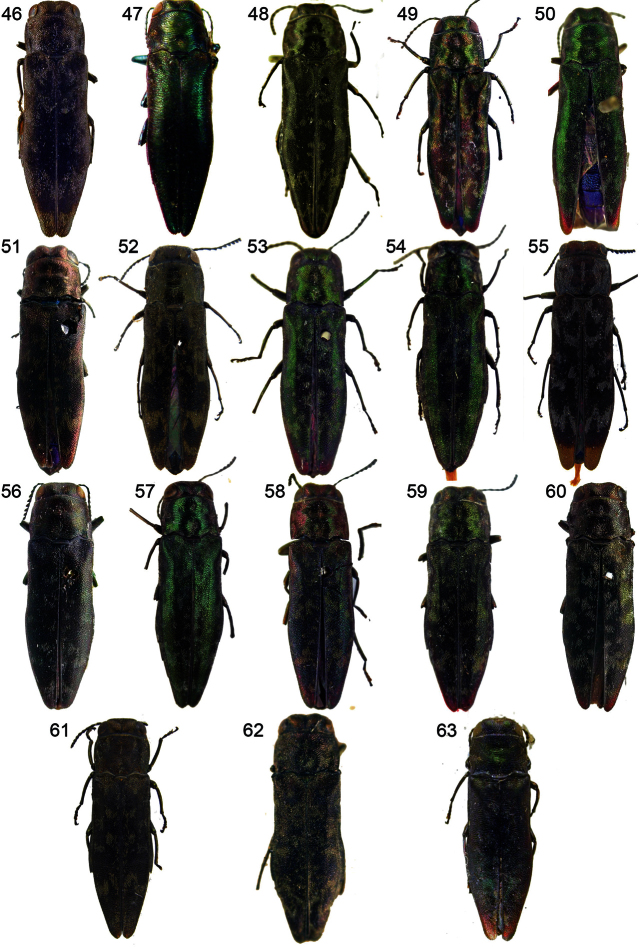
Types of *Agrilus*: **46** *Agrilus auroapicalis* Kurosawa, 1957 – Holotype **47** *Agrilus biakanus* Curletti, 2006 – Holotype (source: Curletti, 2006) **48** *Agrilus celebicola* Obenberger, 1924 – Lectotype**49** *Agrilus connexus* Kerremans, 1900 – Holotype by monotypy **50** *Agrilus cupricauda* Saunders, 1867 – Lectotype **51** *Agrilus evinadus* Gory & Laporte, 1839 – Lectotype **52** *Agrilus horni* Théry, 1904 – Lectotype **53** *Agrilus korenskyi* Obenberger, 1923 – Lectotype **54** *Agrilus kurandae* Obenberger, 1923 – Lectotype **55** *Agrilus laurenconi* Descarpentries & Villiers, 1963 – Holotype **56** *Agrilus marmoreus* Deyrolle, 1864 – Lectotype **57** *Agrilus nitidus* Kerremans, 1898 – Lectotype **58** *Agrilus nirius* Obenberger, 1924 – Lectotype **59** *Agrilus oblatus* Kerremans, 1900 – Lectotype **60 ***Agrilus occipitalis* (Eschscholtz, 1822) – Lectotype **61** *Agrilus samoensis* Blair, 1928 – Holotype **62** *Agrilus sordidulus* Obenberger, 1916 – Holotype by monotypy **63** *Agrilus tebinganus* Obenberger, 1924 – Lectotype.

##### Diagnosis.

Size: 5.5–9.3 mm. *Agrilus occipitalis* is very variable in the size, color and the shape of body parts. The pygidium varies from arcuate to subangulate (Figs 43-45). From the close *Agrilus horniellus* and *Agrilus sordidulus*, it differs by the characters cited in diagnosis at *Agrilus horniellus* and *Agrilus sordidulus*. See also characters in Appendix.

##### Additional material.

INDONESIA: Java: 1 ♂ (EJCB): “F. H. Doesburg, Java, Samarang”; 1 ♂ (EJCB): “Java Samarang”; 1 ♂ (EJCB): “Java Malang”; 1 (USNM): “L. G. E. Kalshoven, Java 250m, Buitenzorg, ix.1924, NS 176”; 1 (USNM): “Dr. L. J. Toxopeus, Preanger, Java, Bandoeng, 27.xi.31, Djeroek”; 1 (USNM): “Dr. L. J. Toxopeus, Preanger, Java, Bandoeng, xi.1932”; 1 ♂ (EJCB): “Indonesia, Java, Bandung, iv.1993 on Citrus trees”; 8 (EJCB): “Indonesia, Java isl, East Java prov., 6 km SE of Lasem, Celering Mt., 23. I. 1998, St. Jákl leg.”; 1 ♂, 1 ♀ (EJCB): “Indonesia, Java cent., Lasem env. - 4 km E of, Gunung Celering 140 m, 23–24. I. 1998, R. Červenka lgt.”. Kalimantan: 3 (EJCB): “Borneo occ. Pontianak 1901”. Lesser Sunda: 2 ♂, 2 ♀ (EJCB): “Sumbawa Colffs.”; 3 ♂ (EJCB): “W Timor, 350 m, Buraen, 50 km S Kupang, 26.i.-9.ii.2006, S. Jákl leg.”. Sulawesi: 1 (EJCB): “Celebes”; 2 ♂, 1 ♀ (USNM): “Celebes NEJ, Watampone vi 1935, leg. L. E. C. Veen”.Sumatra: 1 ♂ (EJCB): “W Sumatra, 1991”; 1 ♀ (EJCB): “W Sumatra, Solok, Jul 1995”; 1 ♂ (EJCB): “Sumatra, Harau valley, April 1996”; 1 ♂, 4 ♀ (EJCB): “W Sumatra, Harau Valley, 700 m, iv.2004, S. Jákl leg.”; 1 ♂ (EJCB): “Indonesia, W Sumatra, Harau Valley, 500-800 m, ca 20 km N of Payakumbuh, iv-v.2006, S. Jákl leg.”; 3 ♂ (EJCB): “Indonesia, W Sumatra, Harau Valley, 500–800 m, ca 20 km N of Payakumbuh, 5-28.ii.2006, S. Jákl leg.”; 1 ♀ (EJCB): “Indonesia, W Sumatra, Harau Valley, 500-800 m, N of Payakumbuh, iv-v.2006”; 1 ♂ (EJCB): “Indonesia, W Sumatra, Harau Valley, 500-800 m, ca 20 km N of Payakumbuh, iv-v.2006, S. Jákl leg.”; 1 ♂ (EJCB): “Indonesia, W Sumatra, Harau Valley, 500–800 m, ca 20 km N of Payakumbuh, ii.2006, S. Jákl leg.”; 8 (EJCB): “Indonesia, W Sumatra, Mt. Tandikat, 400-600m, ca 25 km N Pariaman, i. 2007 S. Jákl leg.”; 1 ♂ (EJCB): “Indonesia, W Sumatra, Harau Valley, 500–800 m, ca 20 km N of Payakumbuh, v-vi.2007, S. Jákl leg.”; 8 (EJCB): “Indonesia, W Sumatra, Harau Valley, 500-800 m, ca 20 km N of Payakumbuh, v-vi.2007, S. Jákl leg.”; 2 ♂, 2 ♀ (EJCB): “Indonesia, W Sumatra, Harau Valley, 500-800 m, ca 20 km N of Payakumbuh, viii.2009, S. Jákl leg.”. LAOS: Vientiane: cca 55 (EJCB): “Laos centr., 27. IV. -1. V. 1997, 70 km NE Vientiane, Ban Phabat env., 150 m, 18°16.1N, 103°10.9E, E. Jendek & O. Šauša leg.”; 1 ♂ (EJCB): “C Laos, Viang Chan prov. Lao Pako resort, 100 m, 50km NE Vientiane, 2002, M. Štrba leg. 28–30. V. ”; 1 ♂, 1 ♀ (EJCB): “LAOS, Vientiane prov., Lao Pako env. 200 m, 55 km NE Vientiane, 1–4.v.2004, F. & L. Kantner leg.”; 1 ♀ (EJCB): “Laos centr., Viang Chan pr., Ban PA Kho resort, ca.50 km, NE of Vientiane, ~90 m, 18°10'N, 102°52'E, 9.–14.vi.2007, M. Štrba leg.”. MALAYSIA: Johor: 1 (EJCB): “Malaysia: Pahang, Tioman island, 2 km N Ayer Batang, 18.7.1993, leg. Schuh”. Pahang: 1 ♀ (EJCB): “Malaysia-W, Pahang pr., 30km E Ipoh, 1500m, Cameron Highlands, Tanah Rata, 20.ii.-3.iii.1998, P. Čechovský leg.”; 1 ♀ (EJCB): “Malaysia-W, Pahang pr., 30km E Ipoh, 1500m, Cameron Highlands, Tanah Rata, 21-24.vi.2001, P. Čechovský leg.”; Perak: 1 ♀ (EJCB): “Malaysia-W, Perak 900m, 40km SE Ipoh, 4°25'N, 101°23'E, Cameron Highlands, Ringlet, M.Říha leg. 25.iv.–5.v.2001”. NORTHERN MARIANA ISLANDS: 13 (USNM): “Mariana Isls.: Saipan Island, Aug. 20, 1944, David G. Hall”; 38 (USNM): “Lake Hagoya, Tinian Is., VI-10-46 \ Oakley 526 on Citrus leaves”; 2 (USNM): “Chalon [?]avlav, Saipan, vi-19-46, Oakley 734, on orange leaves”; 1 (USNM): “Rota Rota, vi-23-46, Townes”; 1 (USNM): “Vlig Bay, Guam, 22-i-48, Mechler \ with Annona recticulata 48-2448”; 5 (USNM): “Chalon Lavlau, Saipan, vi-19-46, Oakley 734, on orange leaves”; 6 (USNM): “Marpo Valley, Tinian Isl., vi-9-46, Oakley 520, on sour orange leaves”. PAPUA NEW GUINEA: 1 ♂ (CBCS): “New Guinea, Sideia island, Sideia Mission, 28 Dec. 1988, Leg. G. Hangay”. PHILIPPINES: Luzon isl. group: 22 (USNM): “Manilla PI, in citrus branches, CollnRC McGregor”; 3 (USNM): “Malinao, Tayabas, Baker”; 1 (USNM): “Manila, PI, CollnRC McGregor”; 1 (USNM): “Mt. Makiling, Luzon, Baker,”; 1 ♂ (EJCB): “Mindoro”; 6 (USNM): “Santo Tomas, Batangas, Luzon”; 8 (USNM): “Manile, Philippines”; 1 (USNM): “Luzon, P. I. , Montalban”; 1 (USNM): “Los Banos, Philippine Is., vi-vii-17”; 3 (USNM): “Manila P. I. , VI-24, R. C. Mc. Gregor”; 5 (USNM): “Manila P. I. , April 24, R. C. Mc. Gregor”; 1 (USNM): “Manila P. I. , V-24, R. C. Mc. Gregor”; 22 (USNM): “in Citrus wood, Manila, P. I. , 8.17.25, FC Brosius”; 1 (USNM): “Los Banos, Luzon PI, X.1945, Bmalkin”; 1 ♀ (EJCB): “Mt. Maquiling Philippines, elev. 50m, 28-II-48, R. Afenir”; 2 ♂, 1 ♀ (EJCB): “Lipa, Batangas, elev. 100m, 10-VIII-1948, Bigornia, A.”;Mindanao isl. group: 2 (USNM): “Zamboanga, Mindanao, Baker”; 1 (USNM): “Zamboanga, Mindanao, Baker”; 3 (USNM): “Iligan, Mindanao”; 2 (USNM): “Surigao, Mindanao, Baker”; 3 (USNM): “Dapitan, Mindanao, Baker”; 1 ♂, 1 ♀ (NMPC): “Dapitan Mindanao Baker”; 6 (USNM): “Davao, Mindanao, Baker”; 1 (USNM): “Diklom, Bukidnon, Mindanao”; 2 ♀ (EJCB): “Philippinen Mindanao”; 15 (USNM): “Butuan, Mindanao, Baker”; 1 ♀ (EJCB): “Mindanao Phillippines 28.vii.1977 M. Sato leg.”; 3 (CBCS): “Philippines, S. E. Mindanao, ix.2009, local collector”. Palawan isl. group: 11 (EJCB): “Philippines, Palawan, 1–21. II. 2000, 800 m, 9°42'N, 118°31E, Salakot waterfalls, E. Jendek leg.”. Visayas islands: 1 (USNM): “Isl. Biliran, Philippines, Baker”; 4 (USNM): “Victoria, occ. Negros, in gardin”; 5 (USNM): “Island Samar, Baker”; 1 ♀ (EJCB): “Victorias, Occ. Negros, 10/26/[19]29”; 6 (USNM): “Calicoan isl., P. I. , x-15-45 FF Bibby 601”; 1 (EJCB): “Masbate P. I. VIII.28 1952 Henry Townes”; 1 ♂ (EJCB): “Malubog, Toledo City, Cebu Is., 12.vi.1986, Hawkeswood T. J. , on steam of Citrus”; 1 (CBCS): “Philippines, Leyte Isl., Mt. Balocaue, vi.2009, local collector”. THAILAND: 1 ♂ (EJCB): “S Thailand Covaz 2.6.1995”, Chiang Mai: 1 ♂ (EJCB): “Thailand 1. VI. 1990 Sansai, Chiang Mai, S. Steinke leg.”. Mae Hong Son: 2 ♂ (EJCB): “Thailand bor., prov. Mae Hong Son, Pai, 24–30. IV. 1997, R. Šigut leg.”. Nakhon Ratchasima: 1 ♂ (MHCB): “Thailand Corat 12.vii.1995, leg. Lehman & Steinke”; 1 ♂, 1 ♀ (EJCB): “Thailand Corat VI.1997”.Yala: 1 ♀ (EJCB): “S Thailand 7-8. V. 1992 Betong, L. Dembický leg.”; 1 ♀ (EJCB): “S Thailand Betong, Gunung Cang dun vill., Yala dist. 25.3.–22.4.93, J. Horák leg.”.VIETNAM: 2 ♀ (EJCB): “Cochinchine”.

##### Adult occurrence:

1–2–3–4–5–6–7–8–9–10–11–12. **Altitude range**: 50–1500 m.

##### Host plant.

*Citrus* sp.: Tan (1925); Clausen (1933); Quayle (1938); Kalshoven (1951 (as *nirius*)); Mühlmann (1954); Balachowsky et al. (1962); Macabasco (1964); Anonymous (1969); Yoshikawa et al. (1969); Hill (2008); *Citrus aurantifolia*: Anonymous (1971); Hawkeswood and Turner (1994 – *Citrus grandis*: Hawkeswood and Turner (1994) – *Citrus microcarpa*: Jackman (1987) – *Citrus sinensis*: Hawkeswood and Turner (1994).

##### Distribution.

AUSTRALIA: Queensland. CAMBODIA: Phnum Penh. CHINA: Taiwan. INDONESIA: Irian Jaya; Java; Kalimantan; Lesser Sunda (incl. West Timor); Maluku; Sulawesi; Sumatra. LAOS: Vientiane. MALAYSIA: Johor; Malaysia Peninsular; Pahang; Perak. NORTHERN MARIANA ISLANDS. PAPUA NEW GUINEA. PHILIPPINES: Luzon isl. group; Mindanao isl. group; Palawan isl. group; Visayas islands. THAILAND: Chiang Mai; Mae Hong Son; Nakhon Ratchasima; Yala. VIE-TNAM: Ba Ria-Vung Tau.

**Remarks**.Obenberger (1936a) cited this species without supportive data also from “Turkestan”. This record needs verification.

#### Agrilus perroti

Descarpentries & Villiers, 1963

http://species-id.net/wiki/Agrilus_perroti

Fig. 2 (habitus) ; Fig. 25 (aedeagus)

Agrilus perroti Descarpentries & Villiers, 1963 (*Agrilus*) Descarpentries and Villiers 1963: 108, 118 (description) – Peng Zhongliang 2002: 269, 281 (characters; new record for China; Fujian) – Jendek 2006: 400 (Palaearctic catalog) – Bellamy 2008: 2233 (world catalog) – Jendek and Grebennikov 2011: 151 (references; types; diagnosis; faunal records; distributional summary; East Asia).

##### Type material.

*Agrilus perroti* Descarpentries & Villiers, 1963. Type locality. Tonkin: Thanh-Moï. Holotype examined by Jendek and Grebennikov (2011).

##### Diagnosis.

See Jendek and Grebennikov (2011) and Appendix.

##### Additional material.

INDIA: West Bengal: 1 ♂, 1 ♀ (MNHN): “British Bootang, Maria Basti, Durel [leg.]”. For further records see: Jendek and Grebennikov (2011).

##### Adult occurrence:

4. **Altitude range**: 400 m.

##### Host plant.

Unknown.

##### Distribution.

CHINA: Fujian; Guangxi; Yunnan. INDIA: West Bengal. VIE-TNAM.

#### Agrilus picturatus sp. n.

urn:lsid:zoobank.org:act:03996C31-A484-4599-8E00-36FB8A1B98C7

http://species-id.net/wiki/Agrilus_picturatus

Fig. 9 (habitus of holotype) ; Fig. 29 (aedeagus)

##### Description.

BODY. Size: 8.4 mm (Holotype).Shape: cuneiform, Build: slender, Color (dorsally): bicolored. HEAD. Medial impression: deep, *Frons*: Shape: flat, *Vertex*: Sculpture elements: rugae, Sculpture shape: semispherical or subparallel, Sculpture density: dense, Sculpture intensity: rough, *Eyes*: Size: moderate, Lower margin: in line or below with antennal socket, Medial orbit: converging ventrally, *Antennae*: Length: long, Width: slender, Serration: from antennomere 4, Antennomere 7-10 (shape): with obvious collum. PRONOTUM. Shape: visually square, Sides: slightly arcuate or straight, Maximal width: at middle, Anterior margin: subequal to posterior, *Anterior lobe*: Size: obvious, Shape: arcuate, Position: projecting beyond anterior angles, *Posterior angles*: Shape: rectangular, Apex: sharp, *Disk*: Impressions: medial and lateral, Medial impression: anteromedial and posteromedial, Lateral impressions (intensity): deep, *Prehumerus*: Development: carinal, Shape: arcuate, Extent: to 1/3 of pronotal length, Anterior end: distant from lateral carina, Posterior end: joined with posterior angle or margin, Arc: moderate, *Lateral carinae*: Interspace: narrow, Convergence: moderately convergent, Junction: absent or present, Narrowest point: at posterior angles. ELYTRA. Color: dichromatic, Alternative color: apical portion, Humeral carina: absent, *Apices*: Arrangement: separate, Shape: arcuate, Modifications: margin obviously denticulate, *Pubescence*: Color: monochromatic, Extent: entire ornamental, rarely entire ornamental with indication of stripes. STERNUM. *Prosternal lobe*: Distal margin: arcuate, *Prosternal process*: Shape: dilated, rarely subparallel, Sides: straight, Angles: obtuse, Angles (tips): blunt, Disc: flat, Projection (extend): distinctly beyond angles, *Mesosternum*: Mesosternal projection: flat. ABDOMEN. *Pygidium*: Apical margin: with shortly projecting carina, *Last ventrite*: Disk: with medial carinula, *Sternal groove*: Extent: on all ventrites or on three apical ventrites, Shape on the apex of last ventrite: arcuate. LEGS. *Metatarsus*: Size to metatibia: about as long or longer than metatibia, *Tarsomere 1*: Size to following tarsomeres: subequal or longer than 2-4. GENITALIA. *Aedeagus* (Fig. 29): Symmetry: symmetric, Shape: widest in basal part, Modifications: apex of medial lobe sharply pointed.

##### Diagnosis.

From the very similar *Agrilus pseudoambiguus* sp. n., it differs by having the pronotum more elongate with sides almost straight; by having an obvious pronotal lobe and by the elytral apices being distinctly denticulate. See also Appendix.

##### Type locality.

Thailand, Sakon Nakhon province, Phu Phane National Park, 17°07'30"N, 104°01'E, altitude 350 m.

##### Type material.

Holotype (Fig. 9), ♂, (EJCB): “Thai, Sakon Nakhon, Phu Phane Nat.Park, 17°07'30"N, 104°01'E, 350m, ix.2000, local collector”.

##### Adult occurrence:

9. **Altitude range**: 350 m.

##### Host plant.

Unknown.

##### Distribution.

THAILAND: Sakhon Nakhon.

##### Etymology.

The specific name is the Latin adjective *picturatus* (painted). It refers to the elytral pubescence of the species.

#### Agrilus pluvius sp. n.

urn:lsid:zoobank.org:act:4C107D00-2832-487F-B508-2899F45C55AA

http://species-id.net/wiki/Agrilus_pluvius

Fig. 23 (habitus of holotype)

##### Description.

BODY. Size: 12.6 mm (Holotype).Shape: cuneiform, Build: robust, Color (dorsally): unicolored. HEAD. Medial impression: deep, Epistoma: raised above frons, *Vertex*: Sculpture elements: rugae, Sculpture shape: semispherical, Sculpture density: dense, *Eyes*: Size: small, Lower margin: in line or below with antennal socket, Medial orbit: subparallel, *Antennae*: Serration: from antennomere 4. PRONOTUM. Shape: transverse, Sides: slightly arcuate, Maximal width: at middle, Anterior margin: subequal to posterior, *Anterior lobe*: Size: moderate, Shape: arcuate, Width: wide, Position: at level with anterior angles or not reaching level of anterior angles, *Posterior angles*: Shape: obtuse, Apex: sharp, *Disk*: Impressions: medial and lateral, Medial impression: anteromedial and posteromedial, Lateral impressions (intensity): deep, *Prehumerus*: Development: carinal, Shape: bisinuate, Extent: to 1/3 of pronotal length, Anterior end: distant from lateral carina, Posterior end: joined with posterior angle or margin, Arc: moderate, *Lateral carinae*: Interspace: narrow, Convergence: moderately convergent, Junction: present, Narrowest point: at posterior angles. ELYTRA. Color: monochromatic, Humeral carina: absent, *Apices*: Arrangement: separate, Shape: arcuate, *Pubescence*: Color: monochromatic, Density: dense, Extent: entire ornamental. STERNUM. *Prosternal lobe*: Size: large, Distal margin: arcuate or subtruncate, *Prosternal process*: Shape: subparallel, Sides: straight, Angles: obtuse, Angles (tips): blunt, Disc: flat, Projection (extend): distinctly beyond angles, *Mesosternum*: Mesosternal projection: flat. ABDOMEN. Tomentum: present, *Pygidium*: Apical margin: arcuate, *Sternal groove*: Extent: on three apical ventrites, Shape on the apex of last ventrite: arcuately sinuate, Emargination (deepness): very shallow. LEGS. *Metatarsus*: Size to metatibia: about as long or longer than metatibia, *Tarsomere 1*: Size to following tarsomeres: subequal or longer than 2-4. GENITALIA. *Ovipositor*: Shape: square (uritiform).

##### Diagnosis.

*Agrilus pluvius* sp. n. is very distinctive by the large size and it differs from all members of the subgroup by having the apical half of elytra more elongate; by the arcuate apical margin of pygidium and by the distinctly sinuate sternal groove on the apex of last ventrite. See also Appendix.

##### Type locality.

Northeastern India, Meghalaya, southwest of Cherrapunjee, 25°13'–14'N 91°40'E, altitude 900 m.

##### Type material.

Holotype (Fig. 23), ♀, (EJCB): “NE India, Meghalaya, SW of Cherrapunjee, 25°13'–14'N 91°40'E, 900m, 5.–24.v.2005, P. Pacholátko leg.”.

##### Adult occurrence:

5. **Altitude range**: 900 m.

##### Host plant.

Unknown.

##### Distribution.

INDIA: Meghalaya.

##### Etymology.

The specific name is Latin adjective *pluvius* (rainy). It refers to the type locality which is known for the highest precipitation in the world.

#### Agrilus pseudoambiguus sp. n.

urn:lsid:zoobank.org:act:9A413050-3A7B-480A-9990-AE8CBA32019D

http://species-id.net/wiki/Agrilus_pseudoambiguus

Fig. 10 (habitus of holotype) ; Fig. 30 (aedeagus)

##### Description.

BODY. Size: 5.9–8.1 mm (Holotype 8.1 mm).Shape: cuneiform, Color (dorsally): bicolored, rarely unicolored, Sexual modifications in male: not apparent. HEAD. Medial impression: deep, Epistoma: raised above frons, *Vertex*: Shape: markedly convex, Sculpture elements: rugae, Sculpture shape: semispherical or subparallel, Sculpture density: dense, *Eyes*: Size: small, Lower margin: in line or below with antennal socket, Medial orbit: converging ventrally, *Antennae*: Length: long, Width: slender, Serration: from antennomere 4, Antennomere 7-10 (shape): with obvious collum. PRONOTUM. Shape: visually square, Sides: slightly arcuate, rarely subangulate, Maximal width: at middle, Anterior margin: subequal to posterior or wider than posterior, *Anterior lobe*: Size: absent or vague, rarely moderate, Shape: arcuate, Position: at level with anterior angles, *Posterior angles*: Shape: rectangular, rarely acute, rarely obtuse, Apex: sharp, *Disk*: Impressions: medial and lateral, Medial impression: anteromedial and posteromedial, Lateral impressions (intensity): deep, Lateral impression (size): narrow, *Prehumerus*: Development: carinal, Shape: arcuate, Extent: to 1/3 of pronotal length, Anterior end: distant from lateral carina, Posterior end: joined with posterior angle or margin, Arc: moderate or weak, *Lateral carinae*: Convergence: moderately convergent, Junction: absent or present, Narrowest point: at posterior angles. ELYTRA. Color: dichromatic, Alternative color: apical portion, *Apices*: Arrangement: separate, Shape: arcuate, *Pubescence*: Color: monochromatic, Extent: entire ornamental, rarely entire ornamental with indication of stripes. STERNUM. *Prosternal lobe*: Distal margin: arcuate, *Prosternal process*: Shape: dilated or subparallel, Sides: straight, Angles: obtuse, Disc: flat, Projection (extend): distinctly beyond angles, *Mesosternum*: Mesosternal projection: flat. ABDOMEN. *Pygidium*: Apical margin: angulate, *Sternal groove*: Extent: on all ventrites or on three apical ventrites, Shape on the apex of last ventrite: arcuate, rarely arcuately sinuate, Emargination (deepness): very shallow. LEGS. *Metatarsus*: Size to metatibia: somewhat shorter as metatibia, *Tarsomere 1*: Size to following tarsomeres: subequal or longer than 2-4. GENITALIA. *Aedeagus* (Fig. 30): Symmetry: symmetric, Shape: subparallel, Modifications: apex of medial lobe sharply pointed, *Ovipositor*: Shape: markedly elongate.

##### Diagnosis.

From very similar *Agrilus picturatus* sp. n., this new species differs by having the pronotum more transverse with sides slightly arcuate; by absent or vague pronotal lobe and by the smooth or very finely denticulate elytral apices. See also Appendix.

##### Type locality.

Laos, Louang Namtha pr., 21°09'N, 101°19'E, Namtha - Muang Sing, 900–1200 m.

##### Type material.

Holotype (Fig. 10), ♂, (EJCB): “Laos, Louang Namtha pr., 21°09'N, 101°19'E, Namtha - Muang Sing, 5–31.v.1997, 900–1200 m, Vit Kubáň leg.”. Paratypes: 2 paratypes (EJCB) with the same data as holotype. 1 paratype (EJCB): “Laos NE, Hua Phan prov., 20°19'N, 104°25'E, 25 km SE Vieng Xai (by road), Ban Kangpabong env., 14–18.v.2001, D. Hauck leg.”

##### Adult occurrence:

5. **Altitude range**: 900–1200 m.

##### Host plant.

Unknown.

##### Distribution.

LAOS:Houaphan; Louang Namtha.

##### Etymology.

The specific name is derived from Greek prefix *pseudo*- (having the appearance of) and the specific name *ambiguus*; it refers to the similarity of the species to *Agrilus ambiguus*.

#### Agrilus sordidulus

Obenberger, 1916

http://species-id.net/wiki/Agrilus_sordidulus

Fig. 20 (habitus) ; Fig. 37 (aedeagus) ; Fig. 62 (Holotype by monotypy)

Agrilus sordidulus Obenberger, 1916 (*Agrilus*) Obenberger 1916: 34-35 (description) – Obenberger 1936a: 1102 (world catalog) – Bellamy 2008: 2300 (world catalog).

##### Type material.

*Agrilus sordidulus* Obenberger, 1916. Type locality. Ostindien: Trichinopoli. Holotype by monotypy (Fig. 62), (NHMB): “Typus [p] [red label with black border] \ 1220 [h] [blue label] \ Koll. Dr. A. Frh. v. Hoschek [p] Trichinopolis Ind. or. [h] \ Agrilus sordidulus Typ! [h] Det. Obenberger [p]”. Described from 1 specimen.

##### Diagnosis.

Size: 6.1–8.4 mm. *Agrilus sordidulus* can distinguished from the similar *Agrilus occipitalis* and *Agrilus horniellus* by the more robust body with the narrowing apical part of elytra shorter; by the head deeply, medially impressed and by the very deep medial pronotal impressions. See also Appendix.

##### Additional material.

INDIA: Karnataka: 1 (CBCS): “S. Coorg, S. India, Ammatti, 3100 ft, II.1952”. Kerala: 2 ♂ (EJCB): “S India, Kerala, Thekkady Periyar Lake, 9.34 N, 77.10 E, 900–1000 m, 19-27. IV. 1997, Dembický & Pacholátko leg.”. Tamil Nadu: 1 ♂, 8 ♀ (EJCB): “S India, Tamil Nadu, Nilgiri Hills, 15 km SE Kotagiri, near Kunchappanai, alt. 900 m, 13-20. V. 1994, 11°22'N, 76°56'E, Z. Kejval lgt.”; 1 ♂, 3 ♀ (EJCB): “India S, Tamil Nadu, Nilgiris, 15 km SE of Kotagiri, Kunchappanai, 900 m, 11.22 N 76.56 E, 7–22.v.2000, leg. P. Pacholátko”; 1 ♂, 1 ♀ (EJCB): “India S, Tamil Nadu, Nilgiri Hills, 11 km SE of Kotagiri, 1100±100 m, 11.24 N 76.56 E, Kunchappanai, leg. L. Dembický, 3-15.v.2002”.

##### Adult occurrence:

2–4–5. **Altitude range**: 900–1200 m.

##### Host plant.

Unknown.

##### Distribution.

INDIA: Karnataka; Kerala; Tamil Nadu.

#### Agrilus tesselatus sp. n.

urn:lsid:zoobank.org:act:4298A27E-4E89-42F4-A989-AF7CC76DB545

http://species-id.net/wiki/Agrilus_tesselatus

Fig. 1 (habitus of holotype)

##### Description.

BODY. Size: 9.9 mm (Holotype).Shape: subparallel, Build: robust, Posterior tapering part: short with broad apex, Color (dorsally): unicolored. HEAD. Medial impression: deep, Epistoma: raised above frons, *Frons*: Shape: markedly convex, *Vertex*: Shape: markedly convex, Sculpture elements: rugae, Sculpture shape: semispherical, *Eyes*: Size: small, Shape: markedly protruding head outline, Lower margin: in line or below with antennal socket, Medial orbit: converging ventrally, *Antennae*: Length: short, Width: solid, Serration: from antennomere 5, Antennomere 7-10 (shape): without collum, Antennomere 7-10 (length): markedly wider than long. PRONOTUM. Shape: transverse, Sides: straight, Maximal width: at posterior margin, Anterior margin: narrower than posterior or subequal to posterior, *Anterior lobe*: Size: obvious, Position: projecting beyond anterior angles, *Posterior angles*: Shape: obtuse, Apex: blunt, *Disk*: Convexity: strongly convex, Impressions: absent or medial and lateral, Medial impression: anteromedial and posteromedial, Lateral impressions (intensity): shallow, Lateral impression (size): narrow, *Prehumerus*: Development: carinal, Shape: straight, Anterior end: distant from lateral carina, Posterior end: distant from angles and margin, *Lateral carinae*: Convergence: moderately convergent, Junction: present, Narrowest point: at posterior angles, *Scutellum*: Disc: impressed, Scutellar carina: obsolete or absent. ELYTRA. Color: monochromatic, Humeral carina: absent, *Apices*: Arrangement: separate, Shape: subtruncate, Truncation: transverse, *Pubescence*: Color: monochromatic, Extent: entire ornamental. STERNUM. *Prosternal lobe*: Distal margin: angulately emarginate, Delimitation: angulate, Emargination (width): wide, *Prosternal process*: Shape: subparallel, Sides: straight, Angles: rectangular, Angles (tips): blunt, Disc: flat, Projection (extend): distinctly beyond angles, *Mesosternum*: Mesosternal projection: flat. ABDOMEN. Tomentum: absent, *Pygidium*: Apical margin: arcuate, *Sternal groove*: Extent: on apical ventrite, Shape on the apex of last ventrite: angulately sinuate, Emargination (width): markedly wide. LEGS. *Metatarsus*: Size to metatibia: distinctly shorter than metatibia, *Tarsomere 1*: Size to following tarsomeres: longer than 2-3 but shorter than 2-4. GENITALIA. *Ovipositor*: Shape: square (uritiform).

##### Diagnosis.

The very distinctive species which differs from all other members of *Agrilus occipitalis* species-group mainly by characters given for the subgroup definition. See also Appendix.

##### Type locality.

NorthVietnam, Tonkin, Ninh Binh province, Cuc-Phuong national park, 20°18'N, 105°39'00"E.

##### Type material.

Holotype (Fig. 1), ♀, (EJCB): “Vietnam N, Tonkin, Cuc-Phuong nat. park, 2–12. V. 1991, E. Jendek leg.”.

##### Adult occurrence:

5.

##### Host plant.

Unknown.

##### Distribution.

VIETNAM: Ninh Binh.

##### Etymology.

The specific name is the Latin adjective *tesselatus* (checkered). It refers to the elytral pubescence of the species.

#### Agrilus tonkineus

Kerremans, 1895

http://species-id.net/wiki/Agrilus_tonkineus

Fig. 24 (habitus) ; Fig. 40 (aedeagus)

Agrilus tonkineus Kerremans, 1895 (*Agrilus*) Kerremans 1895: 222-223 (description) – Kerremans 1903: 277 (catalog) – Obenberger 1936a: 1105 (world catalog) – Baudon 1963: 53 (faunal record; Laos) – Descarpentries and Villiers 1963: 105, 109 (lectotype designation; characters; faunal records; Tonkin; Laos) – Baudon 1968: 130, 143 (characters in key; faunal records; Laos) – Kurosawa 1974: 3 (characters; notes) – Bellamy 2008: 2330 (world catalog) – Jendek and Grebennikov 2011: 206 (synonymy; references; types; diagnosis; faunal records; host plants; distributional summary; East Asia). = *blatteiceps* Bourgoin, 1925 (*Agrilus*) Bourgoin 1925: 131 (description) – Théry 1935b: 15 (synonym of *tonkineus*) – Obenberger 1936a: 1105 (synonym of *tonkineus*) – Baudon 1962: 69 (faunal record; Laos) – Descarpentries and Villiers 1963: 109 (synonym of *tonkineus*) – Bellamy 2008: 2321 (synonym of *tonkineus*; world catalog) – Jendek and Grebennikov 2011: 206 (synonym of *tonkineus*).

##### Type material.

*Agrilus tonkineus* Kerremans, 1895. Type locality. Hanoï. Lectotype designated by Descarpentries and Villiers (1963).

*Agrilus blatteiceps* Bourgoin, 1925. Type locality. Laos: Vien Poukha. Holotype by monotypy examined by Jendek and Grebennikov (2011).

##### Diagnosis.

Size: 7.5–9.5 mm. Very close to *Agrilus mucidus* sp. n. from which it can be distinguished by the pronotum more convex; by the arcuate prehumerus and by the absence of transverse tomentose strip at apical third of elytra. See also Appendix.

##### Additional material.

CHINA: Fujian: 1 ♂ (EJCB): “Shunchang Fujian, 27.iv.1979, Shicheng Ji leg”. Hainan: 1 ♀ (USNM): “Hainan Is, Woh Hau Chuen, E of Nodoa, Jul 3, 1929”; 1 ♀ (MNHN): “Hainan, Hu...[illegible], G. Ros leg. 23.v.[19]36”. Yunnan: 1 (IZAS): “Yunnan Xishuangbanna Menghun, 1200-1400m, 28.iv.1958, C. P. Hong leg. [in Chinese]”; 1 (IZAS): “Yunnan Xishuangbanna Mengsong, 1600m, 28.iv.1958, S. Y. Wang leg. [in Chinese]”; 1 (IZAS): “Yunnan Xishuangbanna Damenglong, 650m, 11.iv.1958, L. Y. Zheng leg. [in Chinese]”. For further records see Jendek and Grebennikov (2011).

##### Adult occurrence:

4–5–6–7. **Altitude range**: 420–1600 m.

##### Host plant.

Unknown.

##### Distribution.

CHINA: Fujian; Hainan; Yunnan. LAOS: Borikhamxai; Louang Namtha; Vientiane; Xaignabouri; Xiangkhoang. VIETNAM: Ha Noi; Ha Tay; Hoa Binh.

#### Agrilus trepanatus sp. n.

urn:lsid:zoobank.org:act:46090B44-156C-4387-BADF-35E4F03F81E4

http://species-id.net/wiki/Agrilus_trepanatus

Fig. 14 (habitus of holotype)

##### Description.

BODY. Size: 10.2–12.7 mm (Holotype 12.7 mm).Shape: subparallel, Build: robust, Posterior tapering part: short with broad apex, Color (dorsally): bicolored. HEAD. Medial impression: deep, Epistoma: raised above frons, *Vertex*: Sculpture elements: rugae, Sculpture shape: semispherical, Sculpture density: dense, *Eyes*: Size: small, Lower margin: in line or below with antennal socket, Medial orbit: subparallel, *Antennae*: Length: long, Width: slender, Serration: from antennomere 4, Antennomere 7-10 (shape): with obvious collum. PRONOTUM. Shape: visually square, Sides: slightly arcuate, Maximal width: at middle, Anterior margin: subequal to posterior, *Anterior lobe*: Size: moderate or obvious, Shape: arcuate, Position: at level with anterior angles, *Posterior angles*: Shape: obtuse or rectangular, Apex: sharp, *Disk*: Convexity: strongly convex, Impressions: medial and lateral, Medial impression: anteromedial and posteromedial, *Prehumerus*: Development: carinal, Shape: arcuate or bisinuate, Extent: to 1/2 of pronotal length or to 1/3 of pronotal length, Modifications: with rudiment at anterior angle, Anterior end: distant from lateral carina, Posterior end: joined with posterior angle or margin, Arc: moderate, *Lateral carinae*: Interspace: narrow, Convergence: moderately convergent, Junction: absent, rarely present, Narrowest point: at posterior 1/5-1/4 of marginal carina, Modifications: submarginal carina posteriorly obliterate. ELYTRA. Color: monochromatic, Humeral carina: absent, *Apices*: Arrangement: separate, Shape: arcuate, *Pubescence*: Color: monochromatic, Extent: entire ornamental with indication of stripes. STERNUM. *Prosternal lobe*: Size: large, Distal margin: arcuate, *Prosternal process*: Shape: dilated or subparallel, Sides: straight, Angles: obtuse, Angles (tips): blunt, Disc: flat, Projection (extend): distinctly beyond angles, *Mesosternum*: Mesosternal projection: flat. ABDOMEN. *Pygidium*: Apical margin: arcuate, *Sternal groove*: Extent: on three apical ventrites, Shape on the apex of last ventrite: arcuate or arcuately sinuate, Emargination (deepness): very shallow. LEGS. *Metatarsus*: Size to metatibia: distinctly shorter than metatibia, *Tarsomere 1*: Size to following tarsomeres: subequal or longer than 2–4. GENITALIA. *Ovipositor*: Shape: markedly elongate.

##### Diagnosis.

*Agrilus trepanatus* sp. n. can be distinguished from all species of the group by the large body; by the strikingly bicolor dorsal side and by the head obviously deeply impressed medially. See also Appendix.

##### Type locality.

South India, Karnataka state, Coorg district, northeastern Virajpet, 75°50'E, 12°13'N, altitude 500 m.

##### Type material.

Holotype (Fig. 14), ♀, (EJCB): “S-India, Karnataka state, Coorg distr., NE Virajpet, 75°50'E, 12°13'N, ca 500m, 4–8.vi.1999, Z. Kejval & M. Trýzna leg.”. Paratypes: 1 paratype, ♀ (EJCB): “India, Karnataka, 12 km SW Yellapur, 7.vii-14.viii.84, B. Gill FIT 500 m”.

##### Adult occurrence:

6–7–8. **Altitude range**: 500 m.

##### Host plant.

Unknown.

##### Distribution.

INDIA: Karnataka.

##### Etymology.

The specific name is an adjective derived from the Greek verb *trepao* (drill, bore) in Latinized form *trepano*. It refers to the conspicuously impressed head of this species.

#### Agrilus umrongso sp. n.

urn:lsid:zoobank.org:act:A12BB28B-30FB-4AC7-8D0A-99F504EA8221

http://species-id.net/wiki/Agrilus_umrongso

Fig. 3 (habitus of holotype)

##### Description.

BODY. Size: 12 mm (Holotype).Shape: cuneiform, Posterior tapering part: long with narrow apex, Color (dorsally): unicolored. HEAD. Medial impression: deep, Epistoma: raised above frons, *Vertex*: Shape: markedly convex, Sculpture elements: rugae, Sculpture shape: semispherical, Sculpture density: dense, *Eyes*: Size: small, Lower margin: in line or below with antennal socket, Medial orbit: converging ventrally, *Antennae*: Serration: from antennomere 4. PRONOTUM. Shape: transverse, Sides: slightly arcuate, Maximal width: at middle or at posterior margin, Anterior margin: subequal to posterior, *Anterior lobe*: Size: moderate, Shape: arcuate, Position: at level with anterior angles or projecting beyond anterior angles, *Posterior angles*: Shape: obtuse, Apex: sharp, *Disk*: Impressions: medial and lateral, Medial impression: anteromedial and posteromedial, *Prehumerus*: Development: carinal, Shape: bisinuate, Extent: to 1/3 of pronotal length, Anterior end: distant from lateral carina, Posterior end: distant from angles and margin, Arc: weak, *Lateral carinae*: Convergence: moderately convergent, Junction: present, Narrowest point: at posterior angles. ELYTRA. Color: monochromatic, Humeral carina: absent, *Apices*: Arrangement: separate, Shape: arcuate, *Pubescence*: Color: monochromatic, Density: dense, Extent: entire ornamental with indication of stripes. STERNUM. *Prosternal lobe*: Size: large, Distal margin: arcuately emarginate, Emargination (width): narrow, *Prosternal process*: Shape: subparallel, Sides: straight, Angles: obtuse, Angles (tips): blunt, Disc: flat, Projection (extend): distinctly beyond angles, *Mesosternum*: Mesosternal projection: flat. ABDOMEN. Tomentum: present, *Pygidium*: Apical margin: arcuate, *Sternal groove*: Extent: on three apical ventrites, Shape on the apex of last ventrite: arcuate. LEGS. *Metatarsus*: Size to metatibia: distinctly shorter than metatibia, *Tarsomere 1*: Size to following tarsomeres: longer than 2-3 but shorter than 2-4. GENITALIA. *Ovipositor*: Shape: markedly elongate.

##### Diagnosis.

*Agrilus umrongso* sp. n. can be distinguished from the very close *Agrilus perroti* by the head much more deeply impressed medially; by the apically arcuate pygidium and by the distinctly emarginate prosternal lobe. See also Appendix.

##### Type locality.

Northeastern India, Assam, 5 km north of Umrongso, altitude 700 m, 25°27'N, 92°43'E.

##### Type material.

Holotype (Fig. 3), ♀, (EJCB): “NE India, Assam, 1999, 5 km N of Umrongso, 700m, 25°27'N, 92°43'E, 17.–25.v., Dembický & Pacholátko leg.”.

##### Adult occurrence:

5. **Altitude range**: 700 m.

##### Host plant.

Unknown.

##### Distribution.

INDIA: Assam.

##### Etymology.

The specific name is a noun in apposition. It refers to the Umrongso, the type locality of the species.

#### Agrilus yamawakii

Kurosawa, 1957

http://species-id.net/wiki/Agrilus_yamawakii

Fig. 15 (habitus) ; Fig. 34 (aedeagus)

Agrilus yamawakii Kurosawa, 1957 (*Agrilus*) Kurosawa 1957: 192 (description) – Kurosawa 1963: 152 (characters; Japan) – Akiyama 1975: 10 (faunal records; Izu Islands) – Kurosawa 1975: 3 – Akiyama 1980: 85, 87 (faunal record; Japan: Kanagawa) – Tôyama 1985b: 33 (faunal record; Ryukyu Islands) – Tôyama 1985a: 24 (iconography; Japan) – Hirashima 1989: 324 (checklist; Japan) –Li Jingke 1992: 92 (checklist; China: Liaoning) – Morimoto and Tadauchi 1995: 232 (checklist; Japan) – Akiyama and Akiyama 1996: 187 (faunal records; Japan: Honshu) – Akiyama and Ohmomo 1997: 43 (checklist; Japan) – Nonnaizab et al. 1999: 113 (checklist; China: Inner Mongolia) – Hua Li Zhong 2002: 91 (checklist; China: Taiwan) – Mühle 2003: 47 (checklist; Taiwan) – Jendek 2006: 403 (Palaearctic catalog) – Bellamy 2008: 2366 (world catalog) – Jendek and Grebennikov 2011: 232 (references; types; diagnosis; faunal records; host plants; distributional summary; East Asia).

##### Type material.

*Agrilus yamawakii* Kurosawa, 1957. Type locality. Mt. Fukuchiyama, Fukuoka Pref., Kyűshű, Japan. Holotype examined by Jendek and Grebennikov (2011).

##### Diagnosis.

Size: 6.9–11.7 mm. *Agrilus yamawakii* differs from other member of the group by the following combination of characters: the antennae moderately long; the elytral pubescence obsolete at least in proximal part; apical-most part of elytral apices pubescent. See also Appendix.

##### Additional material.

KOREA SOUTH:1 ♂ (MNHN): “Corée, Mirinai, Chass. indigènes”. For further records see Jendek and Grebennikov (2011).

##### Adult occurrence:

5–6–7.

##### Host plant.

*Fagara* (=*Zanthoxylum*) *ailanthoides*; *Fagara mantchurica*: Akiyama and Ohmomo (1997).

##### Distribution.

CHINA: Liaoning; Nei Mongol; Taiwan. JAPAN: Honshu; Kyushu; Ryukyu isl. (Okinawa incl.); Shikoku; Tsushima. KOREA NORTH. KOREA SOUTH.

#### Agrilus zanthoxylumi

Li Meng Lou, 1989

http://species-id.net/wiki/Agrilus_zanthoxylumi

Fig. 4 (habitus) ; Fig. 26 (aedeagus)

Agrilus zanthoxylumi Li Meng Lou, 1989 (*Agrilus*) Li Meng Lou 1989: 60–63 (description) – Zhang Runzi 1988: 16-17 ([Note: no scientific name is cited]; biology) – Li Meng Lou et al. 1990: 34–38 (Hou and Feng are cited as the authors; biology; damage character; spatial distribution; China: Shaanxi) – Zhang Rung Ke and Wang Tong Mu 1992: 402–403 (Hou is cited as the author; characters; biology) – Jendek 2006: 403 (Zhang and Wang are cited as the authors; Palaearctic catalog) – Wu Hai 2006: 236–239 (Hou and Feng are cited as the authors; biology; control methods; China: Shandong) – Löbl and Smetana 2007: 29 (authorship of species corrected to Zhang R. & Wang) – Bellamy 2008: 2367 (Zhang and Wang are cited as the authors; world catalog) – Jendek and Grebennikov 2011: 233 (references; types; diagnosis; faunal records; host plants; distributional summary; East Asia).

##### Type material.

*Agrilus zanthoxylumi* Li Meng Lou, 1989. Type locality. Shaanxi (Baoji, Weinan). Type specimens not found. Described from unknown number of specimens. See also Remarks.

##### Diagnosis.

**S**ee Jendek and Grebennikov (2011) and Appendix.

##### Additional material.

See Jendek and Grebennikov (2011).

##### Adult occurrence:

5–6.

##### Host plant.

*Zanthoxylum*: Li Meng Lou (1989); *Zanthoxylum bungeanum*: Wu Hai (2006).

##### Distribution.

CHINA: Gansu; Hubei; Shaanxi; Shandong; Yunnan; Zhejiang.

**Remarks**.The authorship of the name *zanthoxylumi* had changed several times. Li Meng Lou (1989) attributed the authorship to Hou and Feng, but by presenting characters he unintentionally made this name available. The primary type of *Agrilus zanthoxylumi* has probably not been fixed.

## Supplementary Material

XML Treatment for Agrilus alesi

XML Treatment for Agrilus ambiguus

XML Treatment for Agrilus auriventris

XML Treatment for Agrilus auroapicalis

XML Treatment for Agrilus auroapicalis ishigakianus

XML Treatment for Agrilus biakanus

XML Treatment for Agrilus diversornatus

XML Treatment for Agrilus horniellus

XML Treatment for Agrilus inamoenus

XML Treatment for Agrilus mucidus

XML Treatment for Agrilus nebulosus

XML Treatment for Agrilus occipitalis

XML Treatment for Agrilus perroti

XML Treatment for Agrilus picturatus

XML Treatment for Agrilus pluvius

XML Treatment for Agrilus pseudoambiguus

XML Treatment for Agrilus sordidulus

XML Treatment for Agrilus tesselatus

XML Treatment for Agrilus tonkineus

XML Treatment for Agrilus trepanatus

XML Treatment for Agrilus umrongso

XML Treatment for Agrilus yamawakii

XML Treatment for Agrilus zanthoxylumi

## Appendix I

Character state matrix for species from *Agrilus occipitalis* species-group with the most important diagnostic characters. (doi: 10.3897/zookeys.256.4272.app) File format: Adobe PDF file (pdf).

## References

[B1] Akiyama K (1975) [The buprestids fauna of Izu Islands (Coleoptera, Buprestidae)]. Coleopterists’ News, Tokyo 25/26: 10. [in Japanese]

[B2] Akiyama K (1980) [Four new records of Buprestids–beetles from Kanagawa Prefecture, with notes on the genus Nipponobuprestis and its allied genera (Coleoptera, Buprestidae)]. Natural History Report of Kanagawa 1: 85-87. [in Japanese]

[B3] Akiyama Y Akiyama K (1996) The Buprestid beetles (Coleoptera: Buprestidae) from Hiroshima Prefecture, southwestern Japan. Miscellaneous Reports of the Hiwa Museum for Natural History 34: 181-192. [in Japanese with English subtitle and summary]

[B4] Akiyama K Ohmomo S (1997) A check list of the Japanese Buprestidae. Gekkan–Mushi (Supplement 1), 67 pp.

[B5] Anonymous (1969) Insect pest survey for the year ending 30th june, 1967. Papua New Guinea agricultural jurnal 21 (2): 49-75.

[B6] Anonymous (1971) Insect pest survey for the year ending 30th june, 1968. Papua New Guinea agricultural jurnal 22 (3): 179-201.

[B7] Baer GA (1886) Catalogue des Coléoptères des Iles Philippines. Annales de la Société Entomologique de France (6) 6: 97–200.

[B8] Balachowsky AS Davatchi A Descarpentries A (1962) Famille des Buprestidae, p. 235–300. In: Balachowsky AS (Ed) Entomologie appliquée à l’Agriculture, Traité. Tome I, Coléoptères, Premier Volume, Caraboidea, Staphylinoidea, Hydrophiloidea, Scarabaeoidea, Dascilloidea, Cantharoidea, Bostrychoidea, Cucujoidea, Phytophaga (Cerambycidae et Bruchidae), Paris, Masson et cie éditeurs, XXVII + 564 pp.

[B9] Baudon A (1961) Contribution à l’étude des Buprestides du Laos. Bulletin de la Société Royale des Sciences Naturelles du Laos No. 1: 57-89.

[B10] Baudon A (1962) Contribution à l’étude des Buprestides du Laos. Bulletin de la Société Royale des Sciences Naturelles du Laos No. 4: 65-97.

[B11] Baudon A (1963) Addenda à la liste des Buprestidae du Laos. Bulletin de la Société Royale des Sciences Naturelles du Laos No. 9: 49-74.

[B12] Baudon A (1968) Catalogue commenté des Buprestidae récoltés au Laos. Deuxième Partie. Vientiane, Ministère de l’Information, 190 pp.

[B13] Bellamy CL (1999) Nomenclatural notes in Buprestidae (Coleoptera). Folia Heyrovskyana 7 (1): 1–11.

[B14] Bellamy CL (2002) Coleoptera: Buprestoidea. XII + 492 pp, 4 col. pls. In: Houston WWK (Ed), 2002: Zoological Catalogue of Australia. Vol. 29.5. Melbourne, CSIRO Publishing, Australia, 492 pp.

[B15] Bellamy CL (2008) A world catalogue and bibliography of the jewel beetles (Coleoptera: Buprestoidea). Volume 4, Agrilinae: Agrilina through Trachyini. Sofia, Pensoft, 79, 1932–2684.

[B16] Blair KG (1928) Heteromera, Bostrychoidea, Malacodermata and Buprestidae. In: Insects of Samoa and other Samoan terrestrial arthropoda, Part IV. Coleoptera, Fasc. 2., London, 67–174.

[B17] Bourgoin A (1922) Diagnoses préliminaires de Buprestides (Col.) de l’Indo–Chine française. Bulletin de la Société Entomologique de France 1922: 20-24.

[B18] Bourgoin A (1925) Diagnoses préliminaires de Buprestides nouveaux de l’Indochine française. Bulletin de la Société Entomologique de France 1925: 130-131.

[B19] Carter HJ (1924a) Australian Coleoptera–notes and new species No. iii. Proceedings of the Linnean Society of New South Wales 49: 19-45.

[B20] Carter HJ (1924b) Australian Coleoptera: notes and new species. No. iv. Proceedings of the Linnean Society of New South Wales 49: 521-536.

[B21] Carter HJ (1929) Preface and species list, In: Carter HJ & Théry A: A check list of the Australian Buprestidae With tables and keys to sub–families, tribes, and genera by Andre Thery, correspondant de muséum de Paris; and figures (plates xxxi. to xxxiii.) drawn by Cedric Deane, A. M. I. E. (Aust.). Australian Zoologists 5: 265-304.

[B22] Carter HJ (1940) Australian Buprestidae and the Junk Catalogue. Annals and Magazine of Natural History (11) 6: 380–389. doi: 10.1080/03745481.1940.9723693

[B23] Chûjô M (1970) Coleoptera of the Loo–Choo Archipelago (II). Memoirs of the Faculty of Education Kagawa University Part 2, No 192: 17–21.

[B24] Chûjô M Matuda R (1940) A list of the buprestid–beetles from Kagosima–Districts, southern end of Kyusyu, Japan. Mushi Fukuoka 13: 62-66.

[B25] Clausen CP (1933) The citrus insects of tropical Asia. United States Department of Agriculture, Washington Circular No. 266: 1-35.

[B26] Curletti G (2001) The genus *Agrilus* in Australia (Coleoptera, Buprestidae). Jewel Beetles No. 9: 1–45, figs, maps.

[B27] Curletti G (2006) The genus *Agrilus* Curtis, 1825 in New Guinea and nearest islands (Coleoptera, Buprestidae). Annali del Museo Civico di Storia Naturale Giacomo Doria XCVIII: 157–257.

[B28] Dejean PFMA (1833) Catalogue des Coléoptères de la collection de M. le comte Dejean. Livraison 1, (section 1–12), Paris, Chez Méquignon–Marvis père et fils, p. 1–96.

[B29] Dejean PFMA (1836) Catalogue des Coléoptères de la collection de M. le comte Dejean. Troisième édition, revue, corrigée et augmentée. Livraisons 1–4, Paris, Chez Méquignon–Marvis père et fils, 384 pp.

[B30] Descarpentries A Chûjô M (1968) Coleoptera from Southeast Asia (VI). 2. Family Buprestidae. Memoirs of the Faculty of Education Kagawa University 2 (161): 8-15.

[B31] Descarpentries A Villiers A (1963) Catalogue raisonné des Buprestidae d’Indochine. II. Agrilini, genre *Agrilus* (2e partie). Revue française d'Entomologie 30 (2): 104-119.

[B32] Deyrolle H (1864) Description des Buprestides de la Malaisie recueillis par M. Wallace. Annales de la Société Entomologique de Belgique 8: 1–272, 305–312.

[B33] Eschscholtz JF (1822) Entomographien. Erste Lieferung. Berlin, G. Reimer, 128 + 3 pp, 2 col. pls.

[B34] Eschscholtz JF (1823) II. Entomographien. Erste Lieferung. Mit zwei illuminirten Kupfertafeln. Berlin, Reimer, 186 pp, 2 col. pl.

[B35] Fang Zhigang Wu Hong (2001) A Checklist of Insects from Zhejiang. China Forestry Publishing House, 452 pp. [in Chinese with Latin name index]

[B36] Fisher WS (1921) New Coleoptera from the Philippine Islands. Family Buprestidae, tribe Agrilini. Philippine Journal of Science, Manila 18 (4): 349-447.

[B37] Fisher WS (1926) Fauna Samarensis: Coleoptera, Buprestidae. Philippine Journal of Science, Manila 31 (2): 235-244.

[B38] Fukutomi H Kurihara T (2011) [Host plant record for *Agrilus auroapicalis ishigakianus* Toyama from Iriomotejima, Japan]. Sayabane 1: 27. [in Japanese]

[B39] Gemminger M Harold E von (1869) Catalogus coleopterorum hucusque descriptorum synonymicus et systematicus. Tom. V, Buprestidae, Trixagidae, Monommidae, Eucnemidae, Elateridae, Cebrionidae. Monachii, Sumptu E. H. Gummi, 1347–1608.

[B40] Gory HL (1841) Histoire naturelle et iconographie des insectes coléoptères, Supplément aux buprestides. Tome IV, Livraisons 43–52, P. Duménil, Paris.

[B41] Gory HL Laporte de Castelnau FL (1839) Histoire naturelle et iconographie des insectes coléoptères, publiée par monographies séparées. Suite aux buprestides. Tome II, Paris, P. Duménil, genera paged separately.

[B42] Gray JE (1848) Nomenclature of coleopterous insects in the collection of the British museum. Part III.–Buprestidae. London, E. Newman, 52 pp.

[B43] Guryeva EL (1974) Buprestidae–Zlatki. In: Kryzhanovskii OL (Ed). : Nasekomye i kleshchi–vrediteli sel’skokhozyaistvennykh kul’tur. Tom II, zhestkokrylye. Leningrad, Nauka: 96-112.

[B44] Hawkeswood TJ Turner JR (1994) A note on the biology and behaviour of *Agrilus occipitalis* Eschscholtz (Coleoptera: Buprestidae) in Papua New Guinea. Jewel Beetles 3: 14-18.

[B45] Hill DS (2008) Pests of crops in warmer climates and their control. Springer Netherlands, 704 pp. doi: 10.1007/978-1-4020-6738-9

[B46] Hirashima Y (1989) Buprestidae, p. 320–327. In: Morimoto & Tadauchi (Eds). A check list of Japanese insects. I, Fukoka, Entomological laboratory, Kyushu University Express, XI + 540 pp.

[B47] Hua Li Zhong (2002) List of Chinese Insects. Vol. II. Guangzhou, Zhongshan (Sun Yat–sen) University Press, 612 pp.

[B48] Huangfu Wei Guo Wei Shu Jun Zheng Hong Hai Liu Peng Cheng Huang Wei Shi Zu Hua Chen Xue Xin (2007) Ovarian development of *Agrilus auriventris* Saunders (Coleoptera: Buprestidae). Acta Entomologica Sinica 50 (7): 682-688. [in Chinese with English subtitle]

[B49] Iga M (1955) Buprestidae, p. 73–82. In: Nakane T (Ed) Coloured illustrations of the insects of Japan. Coleoptera. Enlarged and revised edition. Osaka, Hoikusha, 274 pp.[in Japanese with English subtitle]

[B50] Iga M (1962) Buprestidae, p. 73–82. In: Nakane T (Ed) Coloured illustration of the insects of Japan (Coleoptera). Osaka, Hoikusha, 274 pp.[in Japanese with English subtitle]

[B51] ICZN (International Commission on Zoological Nomenclature) (1999) International Code of Zoological Nomenclature. Fourth Edition, adopted by the International Union of Biological Sciences. London, International Trust for Zoological Nomenclature, XXIX + 306 pp.

[B52] Isrigaya T (1963) [On the occurrence and control of citrus flat–headed borer [*Agrilus auriventris*] in Wakayama Prefecture]. Plant Protection 17 (9): 351-355. [in Japanese]

[B53] Jackman JA (1987) A new species of Sambus from Luzon Island, Philippines, with notes on other species in other genera (Buprestidae: Coleoptera). Coleopterists Bulletin 41 (1): 27–33.

[B54] Jakobson GG (1913) Zhuki Rossii i zapadnoi Evropy. Rukovodstvo k’’ opredeleniyu zhukov. Vypusk’’ X–yi. A. F. Devrien’’, S.–Peterburg’’, (2) + p. 721–864, pls 76–83 [Buprestidae: 770–800]. [in Russian]

[B55] Jendek E (1998) Lectotype designations in the Palaearctic and Oriental *Agrilus* species (Coleoptera: Buprestidae) of the Oberthür’s collection in the Muséum national d’Histoire naturelle, Paris. Acta Societatis Zoologicae Bohemicae 62: 315-333.

[B56] Jendek E (2000) Studies in the Palaearctic and Oriental *Agrilus* (Coleoptera, Buprestidae). I. Biológia, Bratislava 55 (5): 501-508.

[B57] Jendek E (2003) Studies in the Palaearctic and Oriental *Agrilus* (Coleoptera, Buprestidae). III. Biológia, Bratislava 58 (2): 179-190.

[B58] Jendek E (2005) Taxonomic and nomenclatural notes on the genus *Agrilus* Curtis (Coleoptera: Buprestidae: Agrilini). Zootaxa 1073: 1-29.

[B59] Jendek E (2006) New nomeclatorial and taxonomic acts and comments. Buprestidae: *Agrilus* p. 60. Catalog: genus *Agrilus* Curtis, 1825, p. 388–403. In: Löbl I, Smetana A (Eds) Catalogue of the Palaearctic Coleoptera, Vol. 3, Stenstrup, Apollo Books, 690 pp.

[B60] Jendek E (2012) Studies in the Palaearctic and Oriental *Agrilus* (Coleoptera, Buprestidae) IV. Zootaxa 3300: 1-19. 10.11646/zootaxa.4560.2.7 31716584

[B61] Jendek E Grebennikov V (2011)*Agrilus* (Coleoptera, Buprestidae) of East Asia. Prague, Jan Farkač, 362 pp.

[B62] Kalshoven LGE (1951) De plagen van de cultuurgewassen in Indonesië. Deel. II, Van Hoeve’s Bandoeng, 1 p + 513–1065 pp +8 col. pls, 281 Figs [in Dutch with English translation of the text for illustrations]

[B63] Kerremans C (1885) Énumération des Buprestides décrits postérieurement au Catalogue de MM. Gemminger & de Harold. 1870–1883. Annales de la Société Entomologique de Belgique 29: 119-157.

[B64] Kerremans C (1892a) Viaggio di Leonardo Fea in Birmania e regioni vicine. XLIX. Buprestides. Annali del Museo Civico di Storia Naturale di Genova Serie 2, 12 (32): 809–832.

[B65] Kerremans C (1892b) Catalogue synonymique des Buprestides decrits de 1758 à 1890. Séance du 5 septembre 1891. Mémoires de la Société Entomologique de Belgique 1: 1-304.

[B66] Kerremans C (1895) Buprestides Indo–Malais. Deuxième partie. Annales de la Société Entomologique de Belgique 39: 192-224.

[B67] Kerremans C (1898) Buprestides nouveaux de l’Australie et des régions voisines. Annales de la Société Entomologique de Belgique 42: 113-182.

[B68] Kerremans C (1900a) Buprestides nouveaux et remarques synonymiques. Annales de la Société Entomologique de Belgique 44: 282-351.

[B69] Kerremans C (1900b) Contribution a l'Etude de la faune entomologique de Sumatra (Côte ouest–Vice–résidence de Païnan). Chasses de M. J. –L. Weyers VI. Buprestides. Mémoires de la Société Entomologique de Belgique 7: 1-60.

[B70] Kerremans C (1903) Coleoptera Serricornia. Fam. Buprestidae, Fasc. 12b, 12c, 12d. In: Wytsman (Ed). Genera Insectorum. Tome II, Fascicules XII–XIV. Verteneuil & Desmet, Bruxelles: 49-338.

[B71] Kurosawa Y (1950) Iconographia Insectorum Japonicorum. [pars Buprestidae p. 3183–3216]. 1031–1186. [in Japanese]

[B72] Kurosawa Y (1956) Buprestidae. In: Esaki T, Ishii T, Kawamura T, Kinoshita S (Eds) Iconographia insectorum Japonicorum, Editio secunda, reformata, Tokyo, Hokuryukan, 1111–1122.

[B73] Kurosawa Y (1957) Buprestid–fauna of Eastern Asia (4). (Coleoptera). Bulletin of the National Science Museum, Tokyo Vol. 3, No. 3 (No. 40): 183–194.

[B74] Kurosawa Y (1963) Buprestidae. 147–156 [Buprestidae], 167–168, 187–188. In: Nakane T, Ohbayashi K, Nomura S, Kurosawa Y (Eds) Iconographia Insectorum Japonicorum. Colore naturali edita. Volumen II (Coleoptera). Tokyo, Hokuryukan, 443 pp, 192 pls. (in Japanese)

[B75] Kurosawa Y (1974) [A comment to the Japanese Buprestidae (10)]. Coleopterists’ News, Tokyo 17/18: 1–4. [in Japanese]

[B76] Kurosawa Y (1975) [A comment to the Japanese Buprestidae (14)]. Coleopterists’ News, Tokyo 27/28: 1–4. [in Japanese]

[B77] Lewis G (1879) A catalogue of Coleoptera from the Japanese archipelago. London, Taylor and Francis, 31 pp.

[B78] Li Jingke (1992) The Coleoptera fauna of Northeast China. Jilin, Education Publishing House, 205 pp.

[B79] Li Meng Lou (1989)*Agrilus zanthoxylumi*, p. 60–63. In: Li Meng Lou, Cao Zhi Min, Wang Pei Xin (Eds): Growth and pest control of Zanthoxylum bungeanum Maxim. Xi’an, Shaanxi Science & Technology Press.

[B80] Li Meng Lou Li Zongming Jia Aiye (1990) Spatial distribution pattern and damage characters of prigkly ash stem girdler. Journal of Nortwestern College of Forestry 5 (1): 34-38. [in Chinese with English subtitle and abstract]

[B81] Lin Gui Rui (2002) List of fruit tree pests of Taiwan and Mainland of China. Wufeng, Taichung, Taiwan, Taiwan Agricultural Research Institute, 847 pp.[in Chinese]

[B82] Löbl I Smetana A (2007) Errata for Volume 3, In: Löbl I Smetana A (Eds). Catalogue of Palaearctic Coleoptera. Volume 4. Elateroidea–Derodontoidea–Bostrychoidea–Cleroidea–Cucujoidea. Apollo Books, Stenstrup: 24-31.

[B83] Macabasco C (1964) The citrus pests and their control. Philippine Journal of Agriculture 26(3–4)): 133-154.

[B84] Mannerheim CG von (1837) Enumeration des Buprestides, et description de quelques nouvelles espèces de cette tribu de la famille des Sternoxes, de la collection de M. Le Comte Mannerheim. Bulletin de la Société Impériale des Naturalistes de Moscou 10: 1-126.

[B85] Miwa Y (1931) A systematic catalogue of Formosan Coleoptera. Entomological laboratory Taihoku Imperial University, Contribution No. 32, Reprinted from the report No. 55 Department of Agriculture, Government Research Institute, Taihoku, Formosa, 359 pp.

[B86] Miwa Y (1940) Buprestidae, In: Hirayama S (Ed) Genshoku Kōchū Zufu, Sanseido, Tokyo, 70–74, 52 pls. [in Japanese]

[B87] Miwa Y Chûjô M (1936) Catalogus Coleopterorum Japonicorum. Buprestidae. Taihoku, Formosa. Taiwan–Konshu–Kenkyusho, 26 + 8 pp index, [corrigenda et addenda separately paginated]. [in Japanese]

[B88] Miwa Y Chûjô M (1940) An iconography of some new and rare species of Buprestidae from the Japanese Empire. Nippon no Kôchû, Tokyo 3 (2): 53–74, 1 pl. (in Japanese and English)

[B89] Morimoto K Tadauchi O (1995) Catalogue of the Japanese wildlife. [Inveretebrata II] Insecta, Tokyo, Japan Wildlife Research Center, 621 pp.[in Japanese]

[B90] Mühle H (2003) Taiwanese buprestids (Coleoptera, Buprestidae). Journal of the Zoological Society Wallacea 1: 43-48.

[B91] Mühlmann H (1954) Buprestidae, Prachtkäfer, Flatheaded borer, In: Blunck H (Ed). Handbuch der Pflanzenkrankheiten. Fünfter Band, Tierische Schädlinge an Nutzpflanzen, 2. Teil, Fünfte neubearbeitete Auflage, Zweite Lieferung, Coleoptera. P. Parey, Berlin und Hamburg: 62-88.

[B92] Naka K Ohashi H (1996) Occurrence and chemical control of the flatheaded citrus borer, Agrilus auriventris (E. Saunders) in Wakayama Prefecture. Proceedings of the Kansai Plant Protection 38: 43–44, Figs [in Japanese with English subtitle]

[B93] Nelson GH Bellamy CL (1993) A clarification of authors and publication dates in the Histoire naturelle et iconographie des insectes coléoptères by F. L. de Laporte & H. L. Gory (Coleoptera: Buprestidae). Giornale Italiano di Entomologia 6: 297-308.

[B94] Nonnaizab Qi Baoying Li Yabai (1999) Insects of Inner Mongolia China, 506 pp. [in Chinese with English subtitle and preface]

[B95] Obenberger J (1914) Agrili generis specierum novarum diagnoses. Časopis České Společnosti Entomologické 11: 41-52.

[B96] Obenberger J (1916) Analecta II. Fam Buprestidae. Neue Beiträge zur systematischen Insektenkunde 1: 30-32.

[B97] Obenberger J (1923) Popisy nových australských krasců. Description of new Australian Buprestidae. Acta Entomologica Musaei Nationalis Pragae 1: 72-81.

[B98] Obenberger J (1924a) Kritische Studien über die Buprestiden (Col.). Archiv für Naturgeschichte 90 (A) Heft 3: 1–171.

[B99] Obenberger J (1924b) Symbolae ad specierum regionis palaearcticae Buprestidarum cognitionem. Jubilejní Sborník Československé Společnosti Entomologické 1924: 6-59.

[B100] Obenberger J (1924c) A study of the Buprestidae, collected by Charles Fuller Baker in Singapore, Borneo and the Philippine Islands. Philippine Journal of Science, Manila 25 (5): 539–660.

[B101] Obenberger J (1926) Buprestidae. In: Winkler A (Ed). Catalogus Coleopterorum regionis palaearcticae. Pars 6, Wien, A. Winkler: 620-663.

[B102] Obenberger J (1931) Příspěvek k poznání javanských krasců. De nonnullis insulae Javae Buprestidarum speciebus. Časopis Československé Společnosti Entomologické 28: 35-39.

[B103] Obenberger J (1935a) Synonymia Agrilorum (Col. Bupr.) II. Časopis Československé Společnosti Entomologické 32: 121.

[B104] Obenberger J (1935b) De regionis palaearcticae generis Agrili speciebus novis (Col. Bupr.). O nových palaearktických druzích krasců z rodu *Agrilus*. Časopis Československé Společnosti Entomologické 32: 161-171.

[B105] Obenberger J (1936a) Buprestidae V., p. 935–1246. In: Junk W & Schenkling S (Eds) Coleopterorum Catalogus, Volumen XIII, Pars 152, Gravenhage, Verlag für Naturwissenschaften, W. Junk, 311 pp.

[B106] Obenberger J (1936b) Synonymia Agrilorum (Col. Bupr.) II. Časopis Československé Společnosti Entomologické 33: 91-92.

[B107] Obenberger J (1940) Ad regionis palaearcticae Buprestidarum cognitionem additamenta. Studie o palaearktických krascích (Col. Bupr.). Acta Musaei nationalis Pragae (Zool. No. 3) 2 B, No. 6: 111-189.

[B108] Ogloblin DA Reichardt AN (1932) Otryad Coleoptera. Zhuki. In: Stackelberg AA (Ed). Spisok vrednykh nasekomykh SSSR i sopredel'Nykh stran. Chast’ I, Vrediteli sel’skogo khozyaistva, Verzeichnis der schädlichen Insekten der paläarktischen region. Teil I. Schädlinge der Landwirtschaft, Tipografya Pechatnaya, Leningrad: 273-327.

[B109] Ohgushi R (1963) Development of ovary and pre–oviposition period of citrus flat–headed borer, *Agrilus auriventris* Saunders under laboratory conditions. Japanese Journal of Applied Entomology and Zoology 7 (2): 92–96. [in Japanese with English subtitle and summary] doi: 10.1303/jjaez.7.92

[B110] Ohgushi R (1966a) Ecological studies on the citrus flat–headed borer, *Agrilus auriventris* Saunders. I. Season of emergence. Japanese Journal of Applied Entomology and Zoology 10: 55–63. [in Japanese with English subtitle and summary] doi: 10.1303/jjaez.10.55

[B111] Ohgushi R (1966b) Studies on the ecology of citrus flat–headed borer [*Agrilus auriventris* Saunders]. 2. Environmental conditions and infestation. Proceeding of Horticultural Society Japan 35: 361–366. [in Japanese with English summary] doi: 10.2503/jjshs.35.361

[B112] Ohgushi R (1978) On an outbreak of the citrus flat–headed borer, *Agrilus auriventris* E. Saunders in Nagasaki prefecture. Research on population ecology 9: 62-74. doi: 10.1007/BF02521397

[B113] Peng Zhongliang (1987) A check list of the buprestid beetles known to China. Journal of Southwest Agricultural University 9 (2): 125–133, 349–364. [in Chinese with English subtitle and summary]

[B114] Peng Zhongliang (1992) Buprestidae. In: Peng Jianwen Liu Youqiao (Eds). Iconography of forest insects in Hunan China. Hunan, Academia Sinica & Hunan Forestry Institute, 1–60, 1–4: 387-408. [in Chinese with English summary]

[B115] Peng Zhongliang (1994) Notes on species of Buprestidae (Coleoptera) new to China. Entomological Journal of East China 3 (1): 17-20. [in Chinese with English summary]

[B116] Peng Zhongliang (2002) Buprestidae, p. 246–281. In: Huang Peikan (Eds) Fauna of Insects in Fujian Province of China Vol. 6: 894 pp.

[B117] Peng Zhongliang Huang Bangkan (1995) Brief notes on diagnosis and biology of citrus buprestid beetle *Agrilus inamoenus* Kerremans (Coleoptera: Buprestidae). Wuyi Science Journal 12: 98–101, Figs [in Chinese with English subtitle and summary]

[B118] Quayle H J (1938) Insects of citrus and other subtropical fruits. Ithaca, New York, Comstock, XI + 583 pp.

[B119] Samouelle G (1819) The entomologist’s useful compendium; or an introduction to the know-ledge of British Insects. T. Boys, London, 496 pp, 12 pls.

[B120] Saunders E (1867) Notes on rare and descriptions of new species of Buprestidae collected by Mr. James Lamb in Penang. Transactions of the Entomological Society of London 5 (7): 509-521.

[B121] Saunders E (1870) Catalogue of the species contained in the genus Buprestis of Linnaeus, previous to its subdivision by Eschscholtz in 1829, referring each to its present genus. London, John van Voorst, V + [I] + 7–37 pp.

[B122] Saunders E (1871) Catalogus Buprestidarum Synonymicus et Systematicus. London, E. W. Janson, XXIII + 171 pp.

[B123] Saunders E (1873) Descriptions of Buprestidae collected in Japan by George Lewis, Esq. Journal of Proceedings of the Linnean Society of London, Zoology 11: 509-523.

[B124] Saunders E (1874) Notes on the Buprestidae collected by Professor Semper in the Philippine lslands; with descriptions of the new species. Transactions of the Entomological Society of London 2: 303-328.

[B125] Schönfeldt H von (1887) Catalog der Coleopteren von Japan mit Angabe der bezüglichen Beschreibungen und der sicher bekannten Fundorte. Jahrbücher des Nassauischen Vereins für Naturkunde 40: 29-204.

[B126] Schultze W (1916) A catalogue of Philippine Coleoptera. Philippine Journal of Science Manila 11 (1): 1-194.

[B127] Shi Fu Ming Liu Yu Shuang Qu Ping (2006) Comparative studies on elytral surface structures of Coraebus and its related genera (Coleoptera, Buprestidae). Acta Zootaxonomica Sinica 31 (4): 723-727.

[B128] Shimizu K Nakashima Y Momiki H (1961) [On the citrus flat–headed borer *Agrilus auriventris* Saunders. 2. Behavior of adult]. Plant Protection, Kyushu 7: 57-59. [in Japanese]

[B129] Tan JP (1925) The citrus bark borer (*Agrilus occipitalis*, Eschsch.). Philippine Agricultural Review 18 (4): 583-584.

[B130] Tanaka K (1928) On the citrus flat–headed borer [*Agrilus auriventris* Saunders], an important insect pest of citrus (Preliminary report). Nogyo oyobi engei [Agriculture & Horticulture], 3, 1437–1444. [in Japanese]

[B131] Ter Minasyan ME (1955) Buprestidae, Bruchidae, Anthribidae, Rhinomaceridae, Attelabidae. In: Arnol’di LV, Medvedev SI, Plavilshchikov NN, Stark VN & Ter Minasyan ME Otryad Coleoptera–Zhestkokrylye ili zhuki. In: Pavlovslii EN (Eds). Vrediteli lesa. Spravochnik. II. Akademiya Nauk SSSR, Zoologicheskii Institut, Leningrad: 425-737. [in Russian]

[B132] Théry A (1904) Buprestides récolts par le Dr Horn a Ceylan. Annales de la Société Entomologique de Belgique 48: 158-167.

[B133] Théry A (1927) Recherches synonymiques sur les Buprestides et descriptions d'Espéces nouvelles (Suite et fin). Bulletin et Annales de la Société Entomologique de Belgique 67(1–2)): 33-48.

[B134] Théry A (1934) Études sur les Buprestides (quatriéme partie). Bulletin et Annales de la Société Entomologique de Belgique 74: 131-151.

[B135] Théry A (1935a) Notes sur quelques Buprestidae (Col.) chinois et description d’une espèce nouvelle. Bulletin de la Société Entomologique de France 40: 132-134.

[B136] Théry A (1935b) Note sur les Buprestides indo–chinois décrits par M. A. Bourgoin. Bulletin de la Société Entomologique de France 40 (1): 14-15.

[B137] Théry A (1935c) Note sur les Buprestides du Museum de Leiden. Zoologische Mededeelingen 18: 241-256.

[B138] Théry A (1936) Notes synonymiques sur les Buprestides (Col.). Bulletin de la Société Entomologique de France 41 (4): 59-61.

[B139] Tôyama M (1985a) Agrilinae. In: Kurosawa Y Hisamatsu S Sasaji H (Eds). Colored illustrations of the Coleoptera of Japan. Vol. III. Osaka, Hoikusha Publishing, first edition: 13-32. [in Japanese]

[B140] Tôyama M (1985b) The buprestid beetles of the subfamily Agrilinae from Japan (Coleoptera, Buprestidae). Elytra 13 (1): 19-47.

[B141] Wei Shu Jun Zheng Hong Hai Huangfu Wei Guo Shi Zu Hua Chen Xue Xin (2006) Division of larval instars of citrus borer, *Agrilus auriventris* Saunders (Coleoptera: Buprestidae). Acta Entomologica Sinica 49 (2): 302-309. [in Chinese with English subtitle and summary]

[B142] Wei Shu Jun Zheng Hong Hai Huangfu Wei Guo Shi Zu Hua Chen Xue Xin (2007) Morphological observation on the immature stages of *Agrilus auriventris* Saunders (Coleoptera: Buprestidae). Acta Entomologica Sinica 50 (1): 79-84. [in Chinese with English subtitle and summary]

[B143] Williams GA (2002) A taxonomic and biogeographic review of the invertebrates of the Central Eastern Rainforest Reserves of Australia (CERRA) World Heritage Area, and adjacent regions. Technical Reports of the Australian Museum 16: 1-208. doi: 10.3853/j.1031-8062.16.2002.1353

[B144] Woo Sheh Ming (1964) Studies on the citrus buprestid beetle, *Agrilus auriventris* Saunders. Acta Phytophylacica Sinica 3 (1): 61-71. [in Chinese with English subtitle and summary]

[B145] Wu Hai (2006) Bionomics and control of *Agrilus zanthoxylumi*. Chinese Bulletin of entomology 43 (2): 236-239. [in Chinese with English summary]

[B146] Yamamoto S Kamimura H Iwasaki M (1961) On the investigation of citrus flat–headed borer [*Agrilus auriventris* Saunders] and gummosis in orchards. Report of Kumamoto Fruit Experiment. Station. No. 1: 1-8. [in Japanese]

[B147] Yoshikawa K Ohgushi R Sakagami S (1969) Preliminary report on entomology of the Osaka City University 5th scientific expedition to Southeast Asia 1966. With descriptions of two new genera of Stenogasterinae wasp. Nature and Life in Southeast Asia 6: 153-182.

[B148] Yuasa H (1932) Family Buprestidae. In: Esaki T, Hori H, Yasumatsu K, Nippon Konchu Zukan. Iconographia insectorum Japonicorum, Editio prima, Tokyo, Hokuryukan, 652–660.

[B149] Yuasa H (1933) On the structure of some Japanese buprestid–larvae, with notes on their life–history. Journal of the Imperial Agricultural Experiment Station, Tokyo 2 (2), 263–282. [in Russian with English summary]

[B150] Yuasa H (1949) Buprestidae. In: Illustrated pocket book of Japanese insects. Tokyo, Hokuryukan, 126–128. [in Japanese with English subtitle]

[B151] Zhang Rung Ke Wang Tong Mu (1992) *Agrilus zanthoxylumi* Hou. In: Xiao Gangrou (Ed). Forest Insects of China. The second edition (Revised & enlarged). Beijing, China forestry publishing house: 402-403.

[B152] Zhang Runzi (1988) [Primary observation on Zanthoxulum–buprestid]. Forest Pest and Disease 4: 16-17. [in Chinese]

[B153] Zheng Hong Hai Wei Shu Jun Huangfu Wei Guo Shi Zu Hua Chen Xue Xin (2006) Spatial distribution pattern of *Agrilus auriventris* Saunders (Coleoptera: Buprestidae). Acta Entomologica Sinica 49 (5): 805-809. [in Chinese with English subtitle and summary]

