# Supplementary material for: Revision of the Agrilus occipitalis species–group (Coleoptera, Buprestidae, Agrilini)
Source: Zookeys. 2013 Jan 3;(256):35–79. doi: 10.3897/zookeys.256.4272 (PMC3650829; doi:10.3897/zookeys.256.4272)
Supplement: Supplementary file 24 — Character state matrix for species from Agrilus occipitalis species-group with the most important diagnostic characters. (doi: 10.3897/zookeys.256.4272.app) File format: Adobe PDF file (pdf). [file ZooKeys-256-035-s001.pdf]

SUBGROUP	SPECIES	BODY		HEAD						PRONOTUM												
		Size		Head (medially)			Antennae (length)			Pronotum (anterior lobe)			Pronotum (maximal width)			Prehumerus (development)			Prehumerus (anterior end)		Prehumerus (posterior end)	
		medium or small (< 10 mm)	large (> 10 mm)	without impression	with moderate impression	deep impression	short	moderate	long	absent or vague	moderate	obvious	at posterior margin	at middle	at anterior margin	carinal	filamentary	obsolete or tubercular	joining marginal carina	distant from marginal carina	joining margin	distant from margin
**tesselatus**	*tesselatus* sp. nov.	●	●			●	●					●	●			●				●		●
**perroti**	*perroti* Descarpentries & Villiers, 1963	○	●		○	●		●	●		●	○	●	●		●				●	●	
	umrongso sp. nov.		●			●		●	●		●		●			●				●		●
	zanthoxylumi Li Meng Lou, 1989	●	○			●			●		●	●	●	○		●				●		●
**auroapicalis**	*auroapicalis* Kurosawa, 1957	●				●		●			○	●		●		●	○		●		●	
	diversornatus Jendek, 2011	●			○	●		●				●	●			●	○	○		●		●
	ishigakianus Tôyama, 1985	●				●		●				●		●		●	●		●		●	
**ambiguus**	*ambiguus* Kerremans, 1895	●			●				●	●	○			●	○	○	●			●	●	
	picturatus sp. nov.	●				●			●			●		●		●				●	●	
	pseudoambiguus sp. nov.	●				●			●	●				●		●				●	●	
**auriventris**	*alesi* Obenberger, 1935	●		○	●			●				●		●		●				●	●	
	auriventris Saunders, 1873	●		●	●	●			●	○	●		●	○	●	●	○			●	●	
	nebulosus sp. nov.	●			○	●			●	●	●				●	●				●	●	
	trepanatus sp. nov.	○	●			●			●		●	●		●		●				●	●	
	yamawakii Kurosawa, 1957	●	●	●	●			●		○	●			○	●	●	●			●	●	
**occipitalis**	*biakanus* Curletti, 2006	●			●		?	?	?		●	●		●		●			●		?	?
	horniellus Obenberger, 1935	●			●				●	●	●			●		●	●			●	●	
	occipitalis (Eschscholtz, 1822)	●		○	●	●			●	●	●	○		●		●	●			●	●	
	sordidulus Obenberger, 1916	●				●		●	●	●	○			●		●		○		●	●	
**inamoenus**	*inamoenus* Kerremans, 1892	●	○			●	●			●			●	●				●		●	●	
	mucidus sp. nov.	●	●			●			●		●			●		○	●			●	●	
	pluvius sp. nov.		●			●		●	●		●		●			●				●	●	
	tonkineus Kerremans, 1895	●	○			●			●	●	●			●		●	○			●	●	

SUBGROUP	SPECIES	ELYTRA											STERNUM				
		Elytral pubescence (design)				Elytral apex (pubescence)		Elytral apices (shape)		Elytral apex (color)			Prosternal lobe (anterior margin)		Prosternal process (shape)		
		stripes	stripes and ornament	ornament	absent	glabrous	pubescent	arcuate or subangulate	subtruncate	same as disk	slightly different	strikingly different	arcuate or subtruncate	emarginate	subparallel	dilated	narrowed
**tesselatus**	*tesselatus* sp. nov.			●		●			●	●				●	●		
**perroti**	*perroti* Descarpentries & Villiers, 1963	●	○				●	●		●			●		●		
	umrongso sp. nov.		●				●	●		●				●	●		
	zanthoxylumi Li Meng Lou, 1989		●				●	●		●			●				●
**auroapicalis**	*auroapicalis* Kurosawa, 1957	●	○				●	●				●		●	●		
	diversornatus Jendek, 2011		●				●	●				●		●	●		●
	ishigakianus Tôyama, 1985	●	○				●	●			●			●	●		
**ambiguus**	*ambiguus* Kerremans, 1895		○	●		●		●		●			●		●		
	picturatus sp. nov.		○	●		●		●				●	●		○	●	
	pseudoambiguus sp. nov.		○	●		●		●			●	●	●		●	●	
**auriventris**	*alesi* Obenberger, 1935		○	●		●		●		●	○		●		●		
	auriventris Saunders, 1873		●			●		●		●	○		●			●	
	nebulosus sp. nov.		○	●		●		●		●			●		○	●	
	trepanatus sp. nov.		●			●		●		●			●		●	●	
	yamawakii Kurosawa, 1957		●		○		●	●		●			●		●		
**occipitalis**	*biakanus* Curletti, 2006				●	●		●		?	?	?	●		?	?	●
	horniellus Obenberger, 1935		●	●		●		●		●			●		●		
	occipitalis (Eschscholtz, 1822)		●	●		●		●		○	●	●	●		○	●	
	sordidulus Obenberger, 1916		○	●		●		●		●	○		●			●	
**inamoenus**	*inamoenus* Kerremans, 1892			●		●		●		○	●		●		●		
	mucidus sp. nov.			●		●		●		●	○		●		●		
	pluvius sp. nov.			●		●		●		●			●		●		
	tonkineus Kerremans, 1895		●	●		●		●		●	●		●		○	●	

SUBGROUP	SPECIES	ABDOMEN								GENITALIA				
		Pygidium (apex)			Apex of last ventrite		Groove on apex of last ventrite			Aedeagus			Ovipositor	
		arcuate	angulate or with short protrusion	with long spine	smooth	with medial carinula	arcuate	faint sinuate	deeply sinuate	wider in apical half	subparallel	wider in basal half	square	prolonged
**tesselatus**	*tesselatus* sp. nov.	●			●				●	?	?	?	●	
**perroti**	*perroti* Descarpentries & Villiers, 1963		○	●	●	●	○	●			●			●
	umrongso sp. nov.	●			●		●			?	?	?		●
	zanthoxylumi Li Meng Lou, 1989		●		●	○		●			●			●
**auroapicalis**	*auroapicalis* Kurosawa, 1957	●			●		●			?	?	?		●
	diversornatus Jendek, 2011	●			●		●			?	?	?	?	?
	ishigakianus Tôyama, 1985	●			●		●				●			●
**ambiguus**	*ambiguus* Kerremans, 1895		●	●		●	●	●			●			●
	picturatus sp. nov.		●			●	●					●	?	?
	pseudoambiguus sp. nov.		●		●		●				●			●
**auriventris**	*alesi* Obenberger, 1935			●		●		●				●		●
	auriventris Saunders, 1873		●		●		○	●				●		●
	nebulosus sp. nov.	●			●		●	○			○	●		●
	trepanatus sp. nov.	●			●		●	●		?	?	?		●
	yamawakii Kurosawa, 1957	●			●		●				●			●
**occipitalis**	*biakanus* Curletti, 2006		●		?	?	?	?	?			●	?	?
	horniellus Obenberger, 1935	●			●		●					●		●
	occipitalis (Eschscholtz, 1822)	○	●		○	●	○	●			○	●		●
	sordidulus Obenberger, 1916	●				●	●				●			●
**inamoenus**	*inamoenus* Kerremans, 1892		●		●		●	○		●			●	
	mucidus sp. nov.		●		●		●				○	●	●	
	pluvius sp. nov.	●			●			●		?	?	?	●	
	tonkineus Kerremans, 1895		●		●		●				●		●	

LEGEND: ● present ○ rarely present (empty) absent ? missing data
